# *SAMM50* rs3761472 causes mitochondrial dysfunction and progression of metabolic dysfunction-associated steatotic liver disease

**DOI:** 10.1016/j.molmet.2026.102423

**Published:** 2026-07-17

**Authors:** Suyeon Kim, Young Jin Kim, Nahyun Kim, Uijin Kim, Yi Seul Park, Jun Ho Yun, Jiwon Heo, Hyunwoo Lee, Jiwon Choi, Sinwoo Park, Inhae Jeong, Bong-Jo Kim, Ha Youn Shin

**Affiliations:** 1 Department of Biomedical Science and Engineering, Konkuk University, Seoul, 05029, Republic of Korea; 2 School of Advanced Biotechnology, Konkuk University, Seoul, 05029, Republic of Korea; 3 Division of Genome Science, Department of Precision Medicine, National Institute of Health, Cheongju-si, 28159, Republic of Korea

**Keywords:** SAMM50, Single nucleotide polymorphism, CRISPR/Cas9, Mitochondria, Metabolic dysfunction-associated steatotic liver disease, Genetic biomarker

## Abstract

**Objectives:**

*SAMM50* rs3761472 is associated with metabolic dysfunction-associated steatotic liver disease (MASLD), but its functional consequences in vivo remain unclear. We investigated whether this variant disrupts mitochondrial function and promotes MASLD progression.

**Methods:**

Associations of rs3761472 with MASLD and liver-related traits were evaluated using Korea Biobank Array data. We generated *Samm50* knock-in (KI) mice carrying the D110G substitution corresponding to human rs3761472 using CRISPR/Cas9 and assessed hepatic mitochondrial homeostasis and MASLD-related phenotypes in mice fed a normal diet or a high-fat diet.

**Results:**

In human genetic analyses, rs3761472 was significantly associated with MASLD and higher serum levels of liver injury markers. *Samm50*-KI mice showed reduced hepatic SAMM50 expression, disrupted mitochondrial organization, impaired mitochondrial respiration and ATP production, increased mitochondrial oxidative stress, inflammatory activation, apoptosis, and liver injury. Following high-fat diet feeding, *Samm50*-KI mice exhibited greater hepatic lipid accumulation and liver injury, together with more pronounced insulin resistance and glucose intolerance, than wild-type mice.

**Conclusions:**

Our findings establish rs3761472 as a functional genetic variant linking mitochondrial architecture to metabolic liver disease pathogenesis, with potential relevance as a genetic biomarker for MASLD susceptibility.

## Highlights

• *SAMM50* rs3761472 (D110G), a common MASLD risk variant, is validated in vivo.
• CRISPR knock-in of SAMM50 D110G reduces hepatic SAMM50 expression.
• SAMM50 D110G disrupts mitochondrial homeostasis, increasing ROS and inflammation.
• High-fat diet accelerates MASLD progression in SAMM50 D110G knock-in mice.

## Graphical abstract

**Figure:**
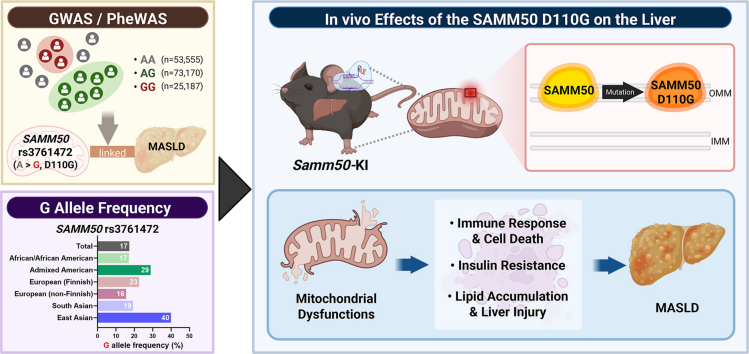

## 1 Introduction

Metabolic dysfunction-associated steatotic liver disease (MASLD) is highly prevalent worldwide and represents a major global health concern. MASLD encompasses a spectrum of liver disorders ranging from simple steatosis to steatohepatitis, fibrosis, and cirrhosis, and may ultimately progress to hepatocellular carcinoma [1]. The development of MASLD is influenced by both environmental and genetic factors, with genetic variants playing a pivotal role in determining individual susceptibility [1,2]. Despite its rising prevalence, most genetic studies have focused on clinical associations, while the molecular mechanisms underlying MASLD pathogenesis remain largely unexplored in in vivo models.

Genome-wide association studies (GWAS) have identified several genetic loci associated with MASLD susceptibility, most notably *PNPLA3*, *TM6SF2*, and *SAMM50* [[3], [4], [5], [6], [7], [8], [9], [10], [11]]. Whereas PNPLA3 and TM6SF2 primarily regulate lipid metabolism, SAMM50 plays a central role in maintaining mitochondrial structure, suggesting that its genetic variant may contribute to MASLD pathogenesis through distinct mechanisms [3,4,12]. *SAMM50* encodes an essential component of the sorting and assembly machinery (SAM) complex in the outer mitochondrial membrane (OMM), which assembles β-barrel proteins and preserves mitochondrial architecture [13,14]. Among four MASLD-associated single-nucleotide polymorphisms (SNPs) in *SAMM50* identified in GWAS, rs3761472 introduces an amino acid change that significantly increases susceptibility to MASLD [[4], [5], [6], [7], [8], [9], [10], [11]]. This variant may impair mitochondrial structure and function, thereby driving disease progression and highlighting novel mitochondrial contributions to MASLD.

These insights align with growing recognition of the importance of mitochondria in MASLD, underscoring their broader involvement in various diseases [15,16]. However, the functional consequences of *SAMM50* rs3761472 have not been validated in in vivo models. To address this gap, we generated a *Samm50* knock-in (KI) mouse harboring the corresponding D110G missense mutation using CRISPR/Cas9. Our findings demonstrate that SAMM50 D110G exacerbates MASLD by impairing mitochondrial function. This study advances understanding of the genetic basis of MASLD and establishes a foundation for biomarker-based risk stratification in individuals carrying the *SAMM50* rs3761472 variant.

## 2 Materials and methods

### 2.1 Genetic association analysis

This study utilizing genotype data was approved by the Institutional Review Board of the Korea Disease Control and Prevention Agency (KDCA), Republic of Korea (IRB no. KDCA-2025-03-02-P-01) and conducted in accordance with the Declaration of Helsinki. Written informed consent was obtained from all participants prior to inclusion in the study. A GWAS of alanine aminotransferase (ALT) levels was previously conducted using 6,949 individuals genotyped using the Korea Biobank Array optimized for the Korean population [17]. Among the loci identified, the nonsynonymous SNP rs3761472 in *SAMM50* was significantly associated with ALT levels (β = 0.0362, P = 7.23 × 10^−6^). In silico prediction tools from dbNSFP v2.7 classified rs3761472 as functionally damaging, with annotations of “Damaging,” or “Disease-causing,” and a CADD score >15 (Table S1). To further investigate, we analyzed genotype dosages for rs3761472 in 151,912 Korean-ancestry individuals from the Korea Biobank Array [18]. Linear regression was performed under an additive genetic model using R (v4.2.0) adjusting for age, age^2^, sex, body-mass index (BMI), recruitment region, and the first four genetic principal components. Finally, we queried the Biobank Japan PheWeb (https://pheweb.jp/) and UK Biobank PheWeb (https://pheweb.org/UKB-TOPMed/) to evaluate associations of rs3761472 with liver-related disease phenotypes [19]. PhastCons and PhyloP scores were retrieved from the UCSC Genome Browser based on multi-species alignment.

### 2.2 In silico protein structure prediction

Protein structure predictions for SAMM50 and its D110G mutant were performed using ColabFold v1.5.5, a Google Colab-based implementation of AlphaFold2. Structural alignment and residue labeling were performed using ChimeraX (version 1.9). Pairwise alignment using the MatchMaker tool quantified structural differences using the root-mean-square deviation (RMSD). The positions of the D110 residue and its mutated counterpart (G110) were identified and visualized using 3D structures.

### 2.3 Mice

All animal experiments were approved by the Institutional Animal Care and Use Committee (IACUC) of Konkuk University (approval no. KU24114) and performed in accordance with institutional guidelines and the ARRIVE guidelines. Wild-type (WT) C57BL/6 mice were purchased from Orient Bio (Seongnam, South Korea). First generation (F1) *Samm50*-KI mice were generated by GEMCRO (formerly Toolgen; Seoul, Korea) using the CRISPR/Cas9 system from a C57BL/6 founder obtained from Orient Bio. To minimize environmental variability, WT and *Samm50*-KI mice were housed under identical environmental conditions throughout the experimental period. Mice were housed in groups of up to five animals per cage in a specific pathogen-free environment, maintained under a 12-hour light/dark cycle at room temperature (20–25 °C) and 30–70% relative humidity, with ad libitum access to food and water. For the experiments, male 16-week-old WT and homozygous KI mice were sacrificed, and serum and liver samples were collected.

### 2.4 Generation of a CRISPR/Cas9–mediated KI mouse line and genotyping

F1 mice with identical genotypes were interbred to produce F2 homozygous mice. Genotyping was performed by polymerase chain reaction (PCR) amplification of genomic DNA isolated from the tail tip, followed by Sanger sequencing. The details of the PCR and sequencing primers are provided in Table S2.

### 2.5 Quantitative reverse transcription-PCR (RT-qPCR)

Total RNA was isolated from the liver tissues of mice using the PureLink RNA Mini Kit (Invitrogen, Carlsbad, CA, USA) according to the manufacturer's instructions. The quantity and quality of isolated RNA were analyzed using a NanoPhotometer N60/N50 spectrophotometer (Implen, München, Germany). cDNA was synthesized from total RNA using the SuperScript III First-Strand Synthesis Supermix (Invitrogen). qPCR was performed on a LightCycler 96 system (Roche, Mannheim, Germany) using SsoAdvanced Universal SYBR Green Supermix (Bio-Rad, Hercules, CA, USA). Gene-specific primers used for qPCR are listed in Table S3. Gene expression levels were normalized to those of glyceraldehyde-3-phosphate dehydrogenase (*Gapdh*), and *Samm50*-KI mRNA levels are presented relative to those of WT.

### 2.6 Western blotting

Western blotting was performed using two distinct protocols. For MTX1, liver lysates were prepared with NP-40 extraction buffer, and protein concentrations were determined using a Bradford assay. Samples were resolved on 4–20% Mini-PROTEAN TGX gels (Bio-Rad) and transferred to PVDF membranes. Membranes were incubated overnight with primary antibodies, washed with TBST, and probed with anti-mouse or anti-rabbit secondary antibodies (Cell Signaling Technology, Danvers, MA, USA) for 1 h. Signals were detected using the Fusion FX system (Viber Lourmat, Collégien, France). For all other proteins, liver lysates were extracted with RIPA buffer containing protease inhibitors (Roche, Basel, Switzerland), and proteins were separated on 10% or 15% SDS-PAGE gels with PageRuler™ Plus markers (Thermo Fisher Scientific, Waltham, MA, USA) or AccuLadder™ 3-color Prestained Protein size marker (Bioneer, Daejeon, South Korea), transferred to nitrocellulose membranes, and detected via enhanced chemiluminescence (Thermo Fisher Scientific) using the ChemiDoc system (Bio-Rad). Antibodies used are listed in Table S4. GAPDH was used as a loading control, and *Samm50*-KI protein levels are presented relative to those of WT.

### 2.7 Measurement of NAD levels and NAD^+^/NADH ratio

NAD^+^ and NADH levels and NAD^+^/NADH ratios were measured using an NAD/NADH Assay Kit (#ab65348; Abcam, Cambridge, MA, USA) as described by the manufacturer. Briefly, tissue samples were homogenized in NADH/NAD extraction buffer and filtered through a 10-kD spin column (#ab93349; Abcam) to remove the enzymes. After completing the assay, the absorbance was measured at 450 nm using a microplate reader (Epoch; BioTek, Winooski, VT, USA). The final NAD^+^ and NADH levels were normalized to the liver protein concentration, as determined by the Bradford assay.

### 2.8 ATP assay

ATP levels in mouse liver were measured using a colorimetric ATP assay kit (#ab83355; Abcam) according to the manufacturer's instructions. Liver tissue lysates were deproteinized with perchloric acid and neutralized with potassium hydroxide. Absorbance was measured at 570 nm using a microplate reader (Epoch; BioTek) to quantify total ATP levels.

### 2.9 Isolation of mouse primary hepatocytes

Primary hepatocytes were isolated from WT and *Samm50*-KI mice using a two-step collagenase perfusion method. Livers were perfused with EGTA-containing buffer followed by digestion with collagenase type IV. Hepatocytes were purified by Percoll gradient centrifugation and resuspended in Medium 199 supplemented with 10% FBS.

### 2.10 Seahorse analysis

Primary mouse hepatocytes were seeded onto Seahorse XF24 cell culture microplates at a density of 60,000 cells per well. Mitochondrial respiration was assessed by measuring the oxygen consumption rate (OCR) using a Seahorse XF24 Extracellular Flux Analyzer (Agilent Technologies, Santa Clara, CA, USA) at the Chronic and Metabolic Disease Research Center (Sookmyung Women's University, Seoul, South Korea). Cells were subjected to a mitochondrial stress test assay with sequential injections of oligomycin (2 μM), FCCP (5 μM), and rotenone/antimycin A (1 μM each). OCR values were normalized to protein content per well.

### 2.11 Measurement of mitochondrial reactive oxygen species (mtROS)

mtROS production in liver tissues was measured using MitoSOX Mitochondrial Superoxide Indicators (#M36008; Invitrogen). Frozen liver tissue (5 μm thick) sections were incubated with MitoSOX Red (5 μM) in the dark at 37 °C for 15 min. The sections were then stained with ProLong Gold Antifade Mountant with DAPI (Invitrogen) in the dark at 25 °C for 1 min. The sections were imaged using a fluorescence microscope (Nikon Eclipse Ts2R; Nikon, Tokyo, Japan) and the fluorescence intensity was quantified using ImageJ software (National Institutes of Health, Bethesda, MD, USA). Three fields of view were imaged for each sample.

### 2.12 Immunofluorescence (IF)

Formalin-fixed paraffin-embedded mouse liver sections (4 μm) were deparaffinized with xylene and ethanol. Antigen retrieval was performed using a TintoRetriever Heat Retrieval System (Bio SB, USA) in 10 mM sodium citrate buffer (pH 6.0) for 10 min. The sections were blocked at 25 °C for 1 h and incubated overnight at 4 °C with an anti-NOS2 antibody (#sc-7271; Santa Cruz Biotechnology, USA). After washing with PBS, tissues were incubated with Alexa Fluor 488-conjugated anti-mouse IgG (#R37120; Invitrogen) for 1 h at 25 °C. Sections were mounted using ProLong Gold Antifade Mountant with DAPI (Invitrogen) and imaged using a fluorescence microscope (Nikon Eclipse Ts2R; Nikon). The fluorescence intensity was quantified using ImageJ software. Three fields per sample were analyzed.

### 2.13 TUNEL assay

DNA fragmentation in apoptotic cells within liver sections was visualized by terminal deoxynucleotidyl transferase-mediated dUTP nick-end labeling (TUNEL) using an in situ BrdU Red DNA Fragmentation Assay Kit (#ab66110; Abcam) according to the manufacturer's instructions. The slides were counterstained with ProLong Gold Antifade Mountant with DAPI (Invitrogen) in the dark at 25 °C for 1 min. TUNEL-positive red fluorescence signals were analyzed using a fluorescence microscope (Nikon Eclipse Ts2R; Nikon). Three fields per sample were imaged for quantification of TUNEL-positive cells.

### 2.14 Biochemical analysis

Mouse serum was obtained by centrifuging whole blood samples at 2,000×*g* for 20 min and collecting the supernatant. Serum levels of ALT, aspartate aminotransferase (AST), total cholesterol (TC), and triglyceride (TG) were measured using an automated dry chemistry analyzer (FUJI DRI-CHEM 7000i, Fujifilm, Tokyo, Japan).

### 2.15 Measurement of serum insulin levels

Serum insulin levels were determined using a rat/mouse insulin ELISA kit (#EZRMI-13K; Millipore, MA, USA) according to the manufacturer's instructions.

### 2.16 Insulin tolerance tests (ITTs) and glucose tolerance tests (GTTs)

ITTs were conducted on mice that had fasted for 6 h; GTTs were performed on mice that had fasted for 16 h. Baseline blood glucose levels in the tail vein blood samples were measured using a glucometer. For ITTs, human insulin was administered via intraperitoneal injection at a dose of 0.75 IU/kg body weight. For GTTs, a 20% glucose solution was administered in the same manner at a dose of 2 g/kg body weight. Glucose levels were measured 15, 30, 45, 60, 90, and 120 min after insulin injection for ITTs and after glucose injection for GTTs.

### 2.17 High-fat diet (HFD)

C57BL/6 and *Samm50*-KI male mice, aged 4 weeks at the start of the study, were used for the experiments. Both WT and *Samm50*-KI mice were maintained on a normal diet (ND) (D12450B; Research Diets, New Jersey, USA) or HFD containing 60 kcal% fat (D12492; Research Diets, New Jersey, USA) for 16 weeks. Body weight was monitored weekly throughout the experiment.

### 2.18 Histopathological analysis

The liver tissues were fixed in 10% formalin, dehydrated, and embedded in paraffin. Tissues were sectioned at a thickness of 4 μm for hematoxylin and eosin (H&E) and Oil Red O staining, which were performed by Labcore (Labcore, Seoul, Korea). Each group included four independent replicates, with at least two fields analyzed per replicate.

### 2.19 Measurement of hepatic TG, TC, and non-esterified fatty acids (NEFA)

Hepatic TG, TC, and NEFA levels were quantified using commercial assay kits according to the manufacturers’ instructions. TG was measured using a PicoSens Triglyceride Assay Kit (#BM-TGR-100; BIOMAX, Seoul, Korea), TC using a Cholesterol Assay Kit (#ab65390; Abcam, Cambridge, UK), and NEFA using a PicoSens Free Fatty Acid Assay Kit (#BM-FFA-100; BIOMAX, Seoul, Korea).

### 2.20 Statistical analysis

Data are presented as mean ± standard error of the mean (SEM) of independent biological replicates. Given the sample size and data distribution, nonparametric Mann–Whitney tests were used for comparisons between two groups. For datasets with n = 3 per group, unpaired two-tailed Student's t-tests were used. For HFD experiments, two-way ANOVA was applied followed by Sidak's post hoc test to correct for multiple comparisons. Detailed information on statistical comparisons is provided in the figure legends.

## 3 Results

### 3.1 Characterization of MASLD-associated variant *SAMM50* rs3761472

**Figure 1:**
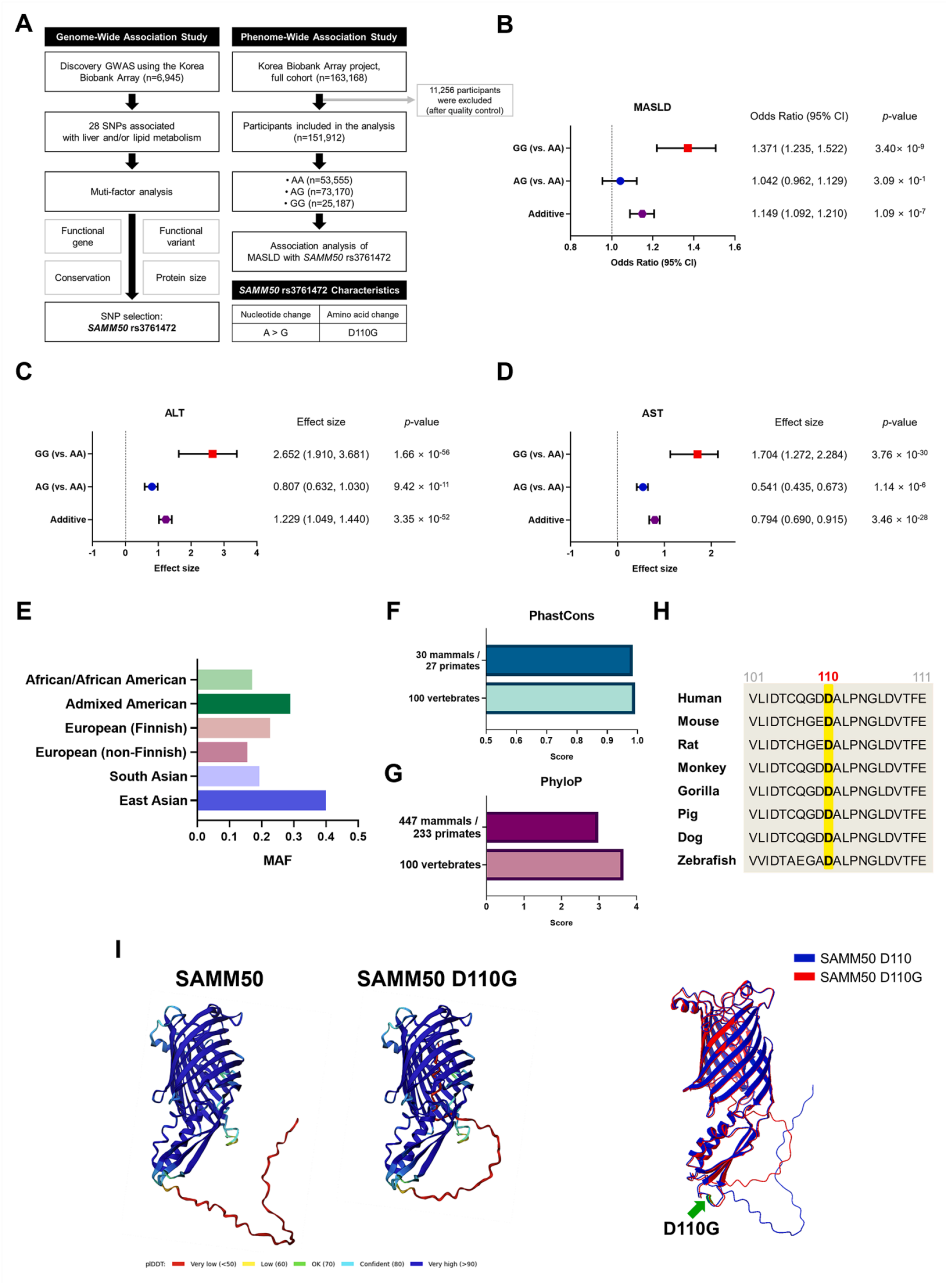
**Characterization of the MASLD-associated *SAMM50* rs3761472 variant. (A)** Study flow chart using the Korea Biobank array. **(B)** Association of rs3761472 genotypes with MASLD risk. **(C**, **D)** Associations of rs3761472 genotypes with serum ALT (C) and AST (D) levels. **(E)** Minor allele frequency of rs3761472 across ethnic groups. **(F**–**H)** Conservation analysis of the rs3761472 locus. Functional prediction scores of PhastCons (F) and PhyloP (G). Multiple sequence alignment showing conservation of residue 110 (highlighted in yellow) across species (H). **(I)** Structural modeling of SAMM50. Left: AlphaFold2-predicted structures of wild-type SAMM50 (D110) and the D110G variant. Right: Structural alignment of the two models, with WT D110 shown in blue and the D110G substitution shown in red. The positions of residues D110 and G110 are indicated by the green arrow.

From the Korean Biobank Array GWAS, we identified *SAMM50* rs3761472 among 28 SNPs associated with liver or lipid metabolism and validated its association with MASLD through a phenome-wide association study (PheWAS) (Figure 1A). The nonsynonymous SNP rs3761472 (A > G; D110G) was predicted to be deleterious by multiple computational tools (Table S1). Homozygous carriers (GG) showed stronger associations with MASLD than the heterozygous carriers (AG) (Fig. 1B), along with increased serum ALT and AST levels (Figure 1C,D). Biobank Japan PheWeb confirmed associations with increased ALT, AST, and higher risk of cirrhosis and hepatocellular carcinoma (Supplementary Fig. 1A) [19]. Consistently, UK Biobank PheWeb revealed associations with chronic liver disease and cirrhosis (Supplementary Fig. 1B). In addition, GWAS Catalog annotations indicated that rs3761472 has been previously associated with liver- and MASLD-related traits, including ALT levels, computed tomography (CT)-derived hepatic fat, and a triglyceride-related metabolic trait (Supplementary Fig. 2). The global MAF of rs3761472 was 17.2%, with enrichment in East Asians (39.9%) and frequent occurrence in Asian MASLD cohorts (Supplementary Fig. 3, Fig. 1E) [[5], [6], [7]]. Its frequency was comparable to *PNPLA3* rs738409 but higher than *TM6SF2* rs58542926 (Supplementary Fig. 3A–J). Cross-species alignment showed conservation of residue 110, supporting functional importance, with high conservation scores (PhastCons >0.98 and PhyloP >2.97 in mammals) (Figure 1F–H).

In silico modeling revealed structural differences between wild-type (WT) SAMM50 and the D110G variant (Fig. 1I). Predicted structures using ColabFold v1.5.5 with AlphaFold2 revealed alterations in loop regions, with an RMSD of 10.1 Å. Such conformational changes suggest potential disruption of protein folding and mitochondrial membrane stability [20,21].

### 3.2 Generation of CRISPR/Cas9–mediated KI mice carrying the *Samm50* rs3761472 genetic variant

**Figure 2:**
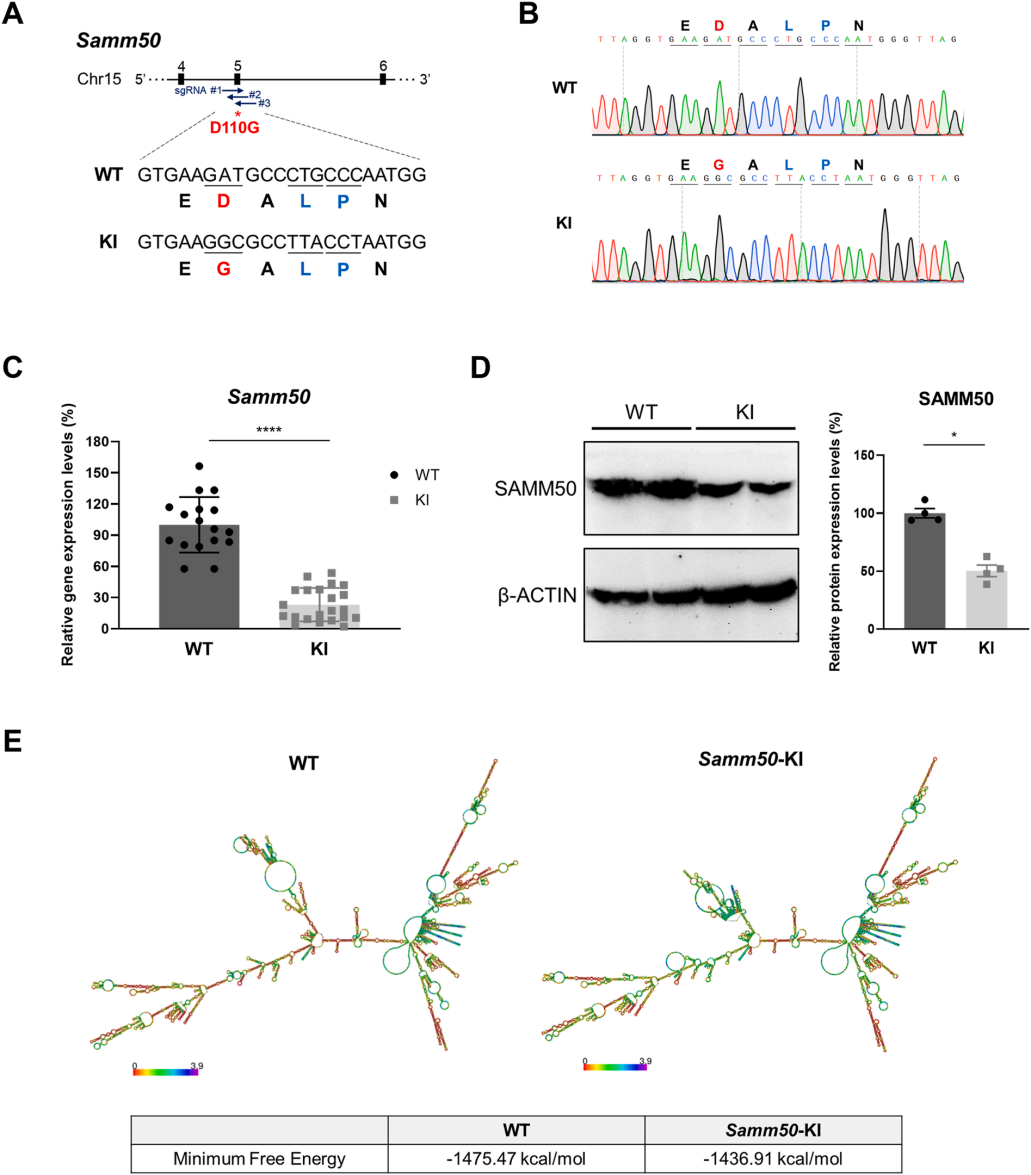
**Generation and validation of *Samm50*-KI mice. (A)** Schematic illustration of sgRNA binding site and sequence changes in *Samm50*-KI mice. The D110 residue was substituted with glycine (G) in exon 5 (red), and silent mutations (blue) were introduced to avoid non-specific Cas9 cutting at NGG sites. **(B)** Sanger sequencing analysis confirming the homozygous mutation of rs3761472 in the *Samm50* gene of KI mice. **(C)** Relative *Samm50* gene expression levels in the livers of WT and *Samm50*-KI mice (WT, n = 17; *Samm50*-KI, n = 22; ∗∗∗∗*p* < 0.0001). **(D)** SAMM50 protein expression levels in WT and *Samm50*-KI mice (WT, n = 4; *Samm50*-KI, n = 4; ∗*p* < 0.05). **(E)** Comparison of centroid secondary structures of *Samm5*0 mRNA in WT and KI mice with minimum free energy.

To investigate the functional consequences of this genetic variant in vivo, we generated *Samm50*-KI mice harboring the D110G mutation using the CRISPR/Cas9 system (Figure 2A, Supplementary Fig. 4A and B). Homozygous KI mice were screened and confirmed by PCR-based genotyping, followed by Sanger sequencing (Fig. 2B). Intriguingly, *Samm5*0 mRNA and SAMM50 protein levels were markedly reduced in *Samm50*-KI mouse livers, indicating the importance of D110 (Figure 2C,D). To further explore how the D110G significantly affected expression levels, we predicted the secondary structure of *Samm5*0 mRNA (http://rna.tbi.univie.ac.at/) [22]. A comparative analysis revealed a distinct alteration in the centroid secondary structure of *Samm50*-KI mRNA relative to WT mRNA (Fig. 2E), which resulted in a 38.56 kcal/mol increase in minimum free energy, indicating reduced mRNA stability [23]. These findings raise the possibility that rs3761472 influences *Samm50* expression by altering mRNA secondary structure, although additional studies are required to directly evaluate its effects on transcript stability.

### 3.3 Impact of SAMM50 D110G mutation on mitochondrial membrane components

**Figure 3:**
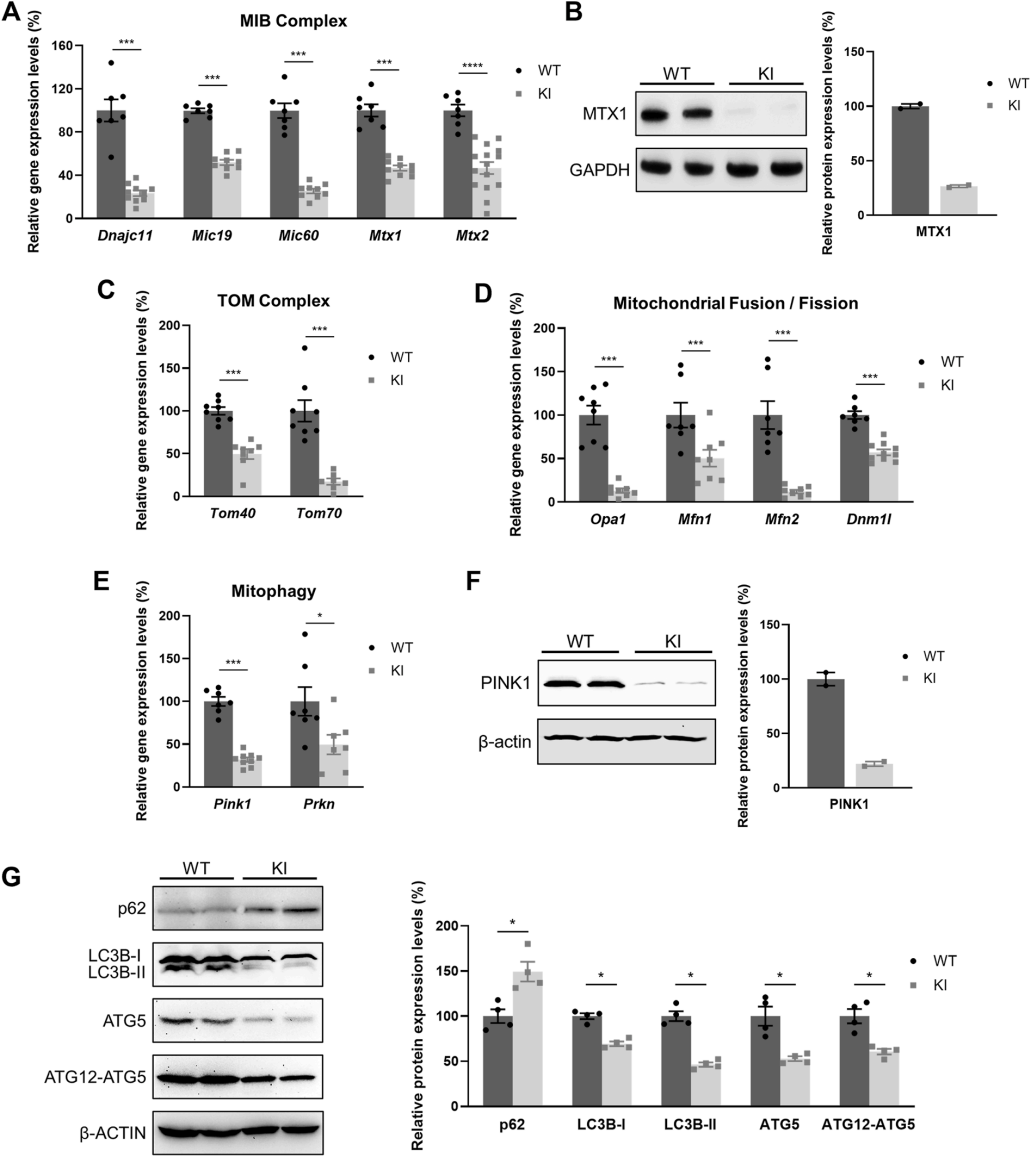
**The impact of the SAMM50 D110G on mitochondrial membrane structure and functional integrity. (A)** Relative mRNA levels of MIB complex genes (WT, n = 7; *Samm50*-KI, n ≥ 9 mice; ∗∗∗*p* < 0.001; ∗∗∗∗*p* < 0.0001). **(B)** MTX1 protein levels (WT, n = 2; *Samm50*-KI, n = 2). **(C)** Relative mRNA levels of TOM complex genes (WT, n = 8; *Samm50*-KI, n ≥ 7 mice; ∗∗∗*p* < 0.001). **(D)** Relative mRNA levels of genes associated with mitochondrial fusion and fission (WT, n = 7; *Samm50*-KI, n ≥ 8 mice; ∗∗∗*p* < 0.001). **(E)** Relative mRNA levels of genes associated with mitophagy (WT, n = 7; *Samm50*-KI, n ≥ 7 mice; ∗*p* < 0.05; ∗∗∗*p* < 0.001). **(F)** PINK1 protein levels (WT, n = 2; *Samm50*-KI, n = 2). **(G)** p62, LC3B, ATG5, and ATG12-ATG5 conjugate protein levels (WT, n = 4; *Samm50*-KI, n = 4; ∗*p* < 0.05).

Several lines of evidence indicate that *SAMM50* knockdown in vitro reduces the expression of mitochondria-associated components such as DNAJC11, MTX1, MTX2, OPA1, and DRP1 [[24], [25], [26]]. To determine whether this effect manifests in vivo, we examined the impact of SAMM50 D110G on key mitochondrial genes maintaining structural integrity in *Samm50*-KI mice. Various protein complexes maintain the double-membrane structure of mitochondria, which comprises the OMM and inner mitochondrial membrane (IMM). These include translocase of the outer membrane (TOM) and SAM complexes, with SAMM50 as a core component. The mitochondrial contact site and cristae organizing system (MICOS) in the IMM forms contact sites that connect the OMM and IMM, and the mitochondrial intermembrane space bridging (MIB) complex encompasses both SAM and MICOS complexes [27,28]. In particular, SAMM50 plays a critical role in maintaining mitochondrial structure through the SAMM50–MIC19–MIC60 axis [13]. We found reductions in the expression of MIB components, including *Dnajc11*, *Mic19*, *Mic60*, *Mtx1*, and *Mtx2* in the livers of *Samm50*-KI (Figure 3A). MTX1 protein levels were also decreased (Figure 3B, Supplementary Fig. 5A). In addition, the TOM members *Tom40* and *Tom70* were downregulated (Fig. 3C).

### 3.4 SAMM50 D110G compromises mitochondrial dynamics and quality control

We further investigated whether the SAMM50 D110G variant affected genes involved in mitochondrial dynamics and quality control, as mitochondria are dynamic organelles that undergo fusion and fission to sustain their function [29]. OPA1 is crucial for fusion of the IMM, whereas mitofusins (MFN1 and MFN2) mediate fusion of the OMM [30]. DRP1 (DNM1L) is a key regulator of mitochondrial fission [31]. We found that the mRNA levels of *Opa1*, *Mfn1*, *Mfn2*, and *Dnm1l* were significantly reduced in the livers of *Samm50*-KI mice (Fig. 3D).

Mitophagy is essential for mitochondrial quality control by removing damaged mitochondria. Under physiological conditions, PINK1 is imported into mitochondria and degraded, but upon mitochondrial damage, it accumulates on the outer membrane, recruits Parkin, and promotes ubiquitination of proteins [32,33]. Ubiquitinated proteins are recognized by p62 and delivered to autophagosomes via LC3B [34]. Notably, certain forms of mitochondrial dysfunction can impair mitophagy, leading to the accumulation of defective mitochondria and increased cellular stress [35]. In *Samm50-*KI mouse livers, the mRNA levels of *Pink1* and *Prkn*, genes critical for mitophagy initiation, were reduced, indicating impaired activation of the mitophagy pathway (Fig. 3E). Consistent with these observations, protein levels of PINK1 were also decreased (Figure 3F, Supplementary Fig. 5B). To further assess whether mitophagic flux was impaired in *Samm50*-KI, we examined key autophagy-related markers, p62, LC3B, ATG5, and the ATG12–ATG5 conjugate. p62 serves as an autophagy adaptor and accumulates when autophagic degradation is impaired, while LC3B and ATG5 are essential for autophagosome formation. In *Samm50*-KI, p62 was increased, while LC3B, ATG5, and the ATG12–ATG5 conjugate were reduced (Fig. 3G). Consistent with these molecular changes, transmission electron microscopy (TEM) analysis revealed mitochondrial ultrastructural abnormalities in the livers of *Samm50*-KI mice (Supplementary Fig. 6). Collectively, these findings indicate that the SAMM50 D110G mutation disrupts mitochondrial dynamics and mitophagy, thereby compromising mitochondrial quality control in the liver.

### 3.5 A single amino acid change, D110G, impairs the mitochondrial function of SAMM50

**Figure 4:**
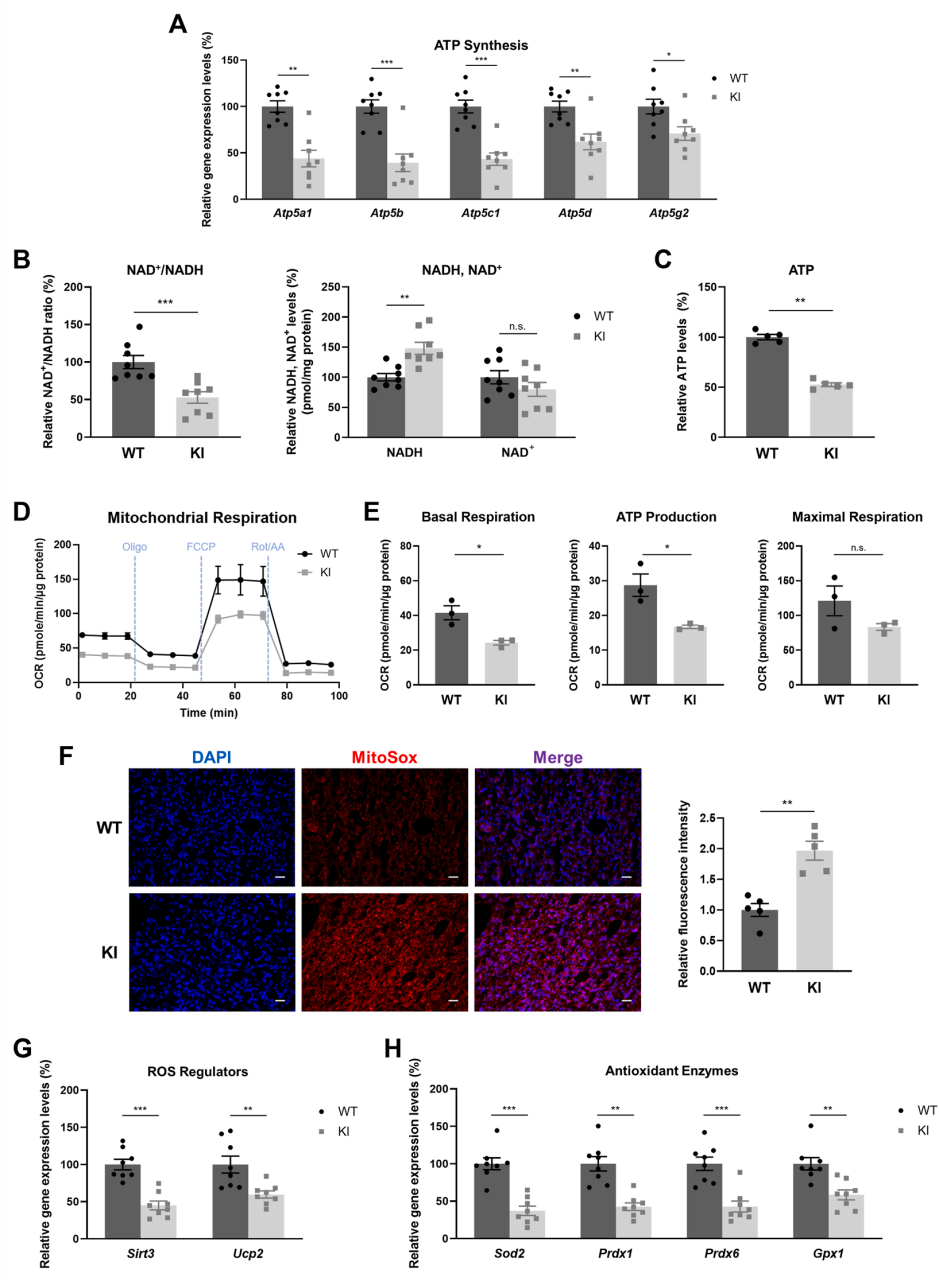
***Samm50*-KI mice display mitochondrial dysfunction. (A)** Relative mRNA levels of genes associated with ATP synthesis (WT, n = 8; *Samm50*-KI, n = 8; ∗*p* < 0.05; ∗∗*p* < 0.01; ∗∗∗*p* < 0.001). **(B)** Left: Comparison of NAD^+^/NADH ratio. Right: NADH and NAD^+^ concentrations in the livers of WT and *Samm50*-KI mice (WT, n = 8; *Samm50*-KI, n = 8; ∗∗*p* < 0.01; ∗∗∗*p* < 0.001; n.s., not significant). **(C)** Hepatic ATP levels (WT, n = 5; *Samm50*-KI, n = 5; ∗∗*p* < 0.01). **(D)** Representative OCR profile of primary hepatocytes isolated from WT and *Samm50*-KI mice using a Seahorse extracellular flux analyzer. **(E)** Quantification of basal respiration, ATP-linked respiration, and maximal respiration (WT, n = 3; *Samm50*-KI, n = 3; ∗*p* < 0.05; n.s., not significant). **(F)** Left: Representative fluorescence images of mitochondrial ROS in liver tissue, stained with MitoSOX Red (× 200 magnification). Scale bars: 100 μm. Right: Quantitative analysis of the relative fluorescence intensity of MitoSOX Red (WT, n = 5; *Samm50*-KI, n = 5; ∗∗*p* < 0.01). **(G, H)** Relative gene expression levels of ROS regulators (G) and antioxidant enzymes (H) (WT, n = 8; *Samm50*-KI, n = 8; ∗∗*p* < 0.01; ∗∗∗*p* < 0.001).

We next investigated the functional consequences of SAMM50 D110G on mitochondrial processes. In *Samm50*-KI mice, expression of genes encoding subunits of Complex V (ATP synthase) was downregulated (Figure 4A), and NAD^+^/NADH ratios decreased resulting from elevated NADH levels (Fig. 4B). Consequently, hepatic ATP levels were markedly reduced by ∼50% compared with WT controls (Fig. 4C). To directly assess mitochondrial respiration, primary hepatocytes isolated from WT and *Samm50*-KI mice were subjected to a mitochondrial stress test assay using a Seahorse XF24 Extracellular Flux Analyzer. *Samm50*-KI hepatocytes exhibited an overall reduction in OCR, accompanied by significantly decreased basal and ATP-linked respiration, and a trend toward reduced maximal respiration (Figure 4D,E). Mitochondrial dysfunction increases mtROS levels owing to impaired electron transport and leakage, triggering mitochondrial damage and cell death [36]. In line with this, mtROS levels were increased in *Samm50*-KI (Fig. 4F), accompanied by a significant reduction in the expression of antioxidant-related genes, including *Sirt3*, *Ucp2*, *Sod2*, *Prdx1*, *Prdx6*, and *Gpx1* (Figure 4G,H). Taken together, SAMM50 D110G inhibits ATP production and elevates mtROS levels, ultimately compromising mitochondrial function.

### 3.6 SAMM50 D110G mutation triggers inflammation and cell death in mouse liver

**Figure 5:**
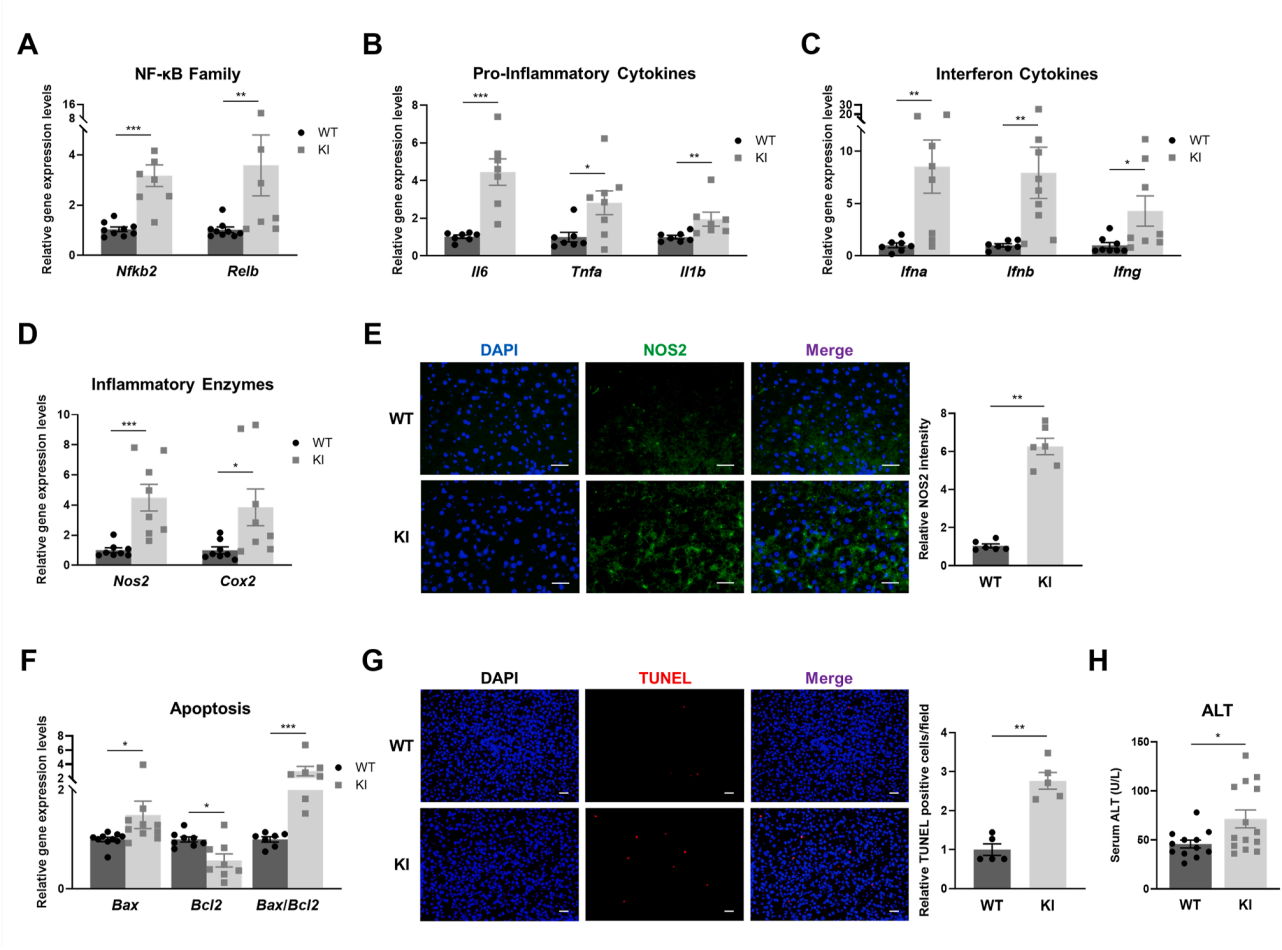
**SAMM50 D110G induces cytokine gene expression and hepatocyte death. (A**–**C)** Relative gene expression levels of NF-κB subunits (A) (WT, n = 7; *Samm50*-KI, n = 8; ∗∗*p* < 0.01; ∗∗∗*p* < 0.001), pro-inflammatory cytokines (B) (WT, n = 7; *Samm50*-KI, n = 7; ∗*p* < 0.05; ∗∗*p* < 0.01; ∗∗∗*p* < 0.001), and type I and type II interferons (C) (WT, n ≥ 7; *Samm50*-KI, n ≥ 7; ∗*p* < 0.05; ∗∗*p* < 0.01). **(D)** Relative gene expression levels of *Nos2* and *Cox2* (WT, n = 8; *Samm50*-KI, n = 8; ∗*p* < 0.05; ∗∗∗*p* < 0.001). **(E)** Left: Immunofluorescence analysis of NOS2 protein (× 400 magnification). Scale bars: 100 μm. Right: Quantitative analysis of the relative fluorescence intensity of NOS2 (WT, n = 6; *Samm50*-KI, n = 6; ∗∗*p* < 0.01). **(F)** Relative gene expression levels of *Bax* and *Bcl-2* and the relative *Bax*/*Bcl-2* ratio (WT, n ≥ 7; *Samm50*-KI, n ≥ 7; ∗*p* < 0.05; ∗∗∗*p* < 0.001). **(G)** Left: Detection of in situ DNA fragmentation in cell nuclei by the TUNEL assay (red) (× 200 magnification). Scale bars: 100 μm. Right: Quantification of the relative number of TUNEL-positive cells per field (WT, n = 5; *Samm50*-KI, n = 5; ∗∗*p* < 0.01). **(H)** Serum ALT levels (WT, n = 12; *Samm50*-KI, n = 14; ∗*p* < 0.05).

As mitochondrial dysfunction induces inflammation through oxidative stress and cytokine release, we evaluated the expression levels of immune-related genes. This analysis showed elevated expression of the NF-κB subunits *Nfkb2* and *RelB*, the pro-inflammatory cytokines *Il6*, *Tnfa*, and *Il1b,* and type I and II interferons *Ifna*, *Ifnb*, and *Ifng* (Figure 5A–C). In addition, increased levels of *Nos2* and *Cox2* mRNA and NOS2 protein were observed in *Samm50*-KI (Figure 5D,E).

Mitochondrial dysfunction and inflammation can accelerate cell death. We measured the expression of *Bax* and *Bcl2*, which regulate mitochondrial membrane integrity. BAX promotes apoptosis by increasing membrane permeability, whereas BCL2 opposes this effect and supports cell survival [37]. In *Samm50*-KI livers, mRNA levels of *Bax* were increased and *Bcl2* reduced, resulting in an elevated *Bax*/*Bcl2* ratio (Fig. 5F), along with a higher number of TUNEL-positive cells (Fig. 5G). Consistent with these findings, ALT levels were significantly elevated in *Samm50*-KI, indicating liver damage (Fig. 5H). Additionally, AST levels showed a ∼30% increase (Supplementary Fig. 7). Taken together, mitochondrial dysfunction caused by the SAMM50 D110G mutation triggers an immune response, leading to cell death and liver damage.

### 3.7 HFD exacerbates MASLD phenotypes in *Samm50*-KI mice

Mitochondrial dysfunction has been reported to precede insulin resistance in MASLD [38]. To evaluate insulin sensitivity and glucose tolerance, we conducted ITTs and GTTs, which revealed mild insulin resistance in *Samm50*-KI mice (Supplementary Fig. 8A–F). We then examined whether *Samm50*-KI mice exhibited MASLD phenotypes. Although MASLD is clinically associated with weight gain and liver pathology, *Samm50*-KI mice showed no significant differences in body weight, liver weight, or liver histology (H&E) compared to WT under standard diet conditions (Supplementary Fig. 9A and B). We also assessed key biochemical markers of MASLD, including serum ALT, AST, TG, and TC. Serum ALT levels were significantly increased in *Samm50*-KI mice, whereas AST, TG, and TC showed a trend toward elevation but did not reach statistical significance (Figure 5H, Supplementary Fig. 7, Supplementary Fig. 9C and D).

**Figure 6:**
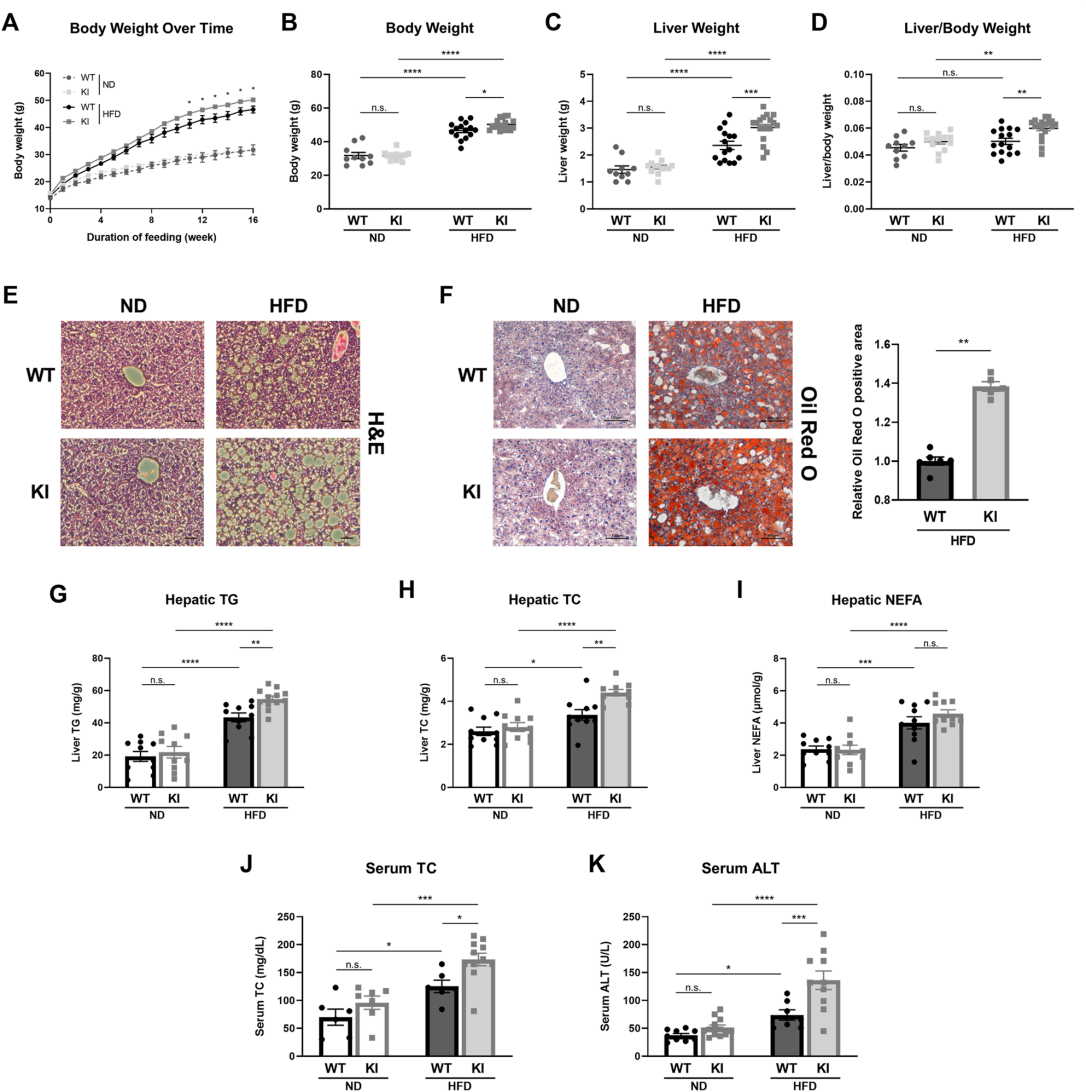
**HFD triggers MASLD-associated clinical signs in *Samm50*-KI mice. (A)** Body weight changes in WT and *Samm50*-KI mice fed either an ND or an HFD over time (WT-ND, n = 10; *Samm50*-KI-ND, n = 13; WT-HFD, n = 15; *Samm50*-KI-HFD, n = 18; ∗*p* < 0.05). **(B**–**D)** Comparison of body weights (B), liver weights (C), and liver-to-body weight ratios (D) in WT and *Samm50*-KI mice after feeding either an ND or HFD (WT-ND, n = 10; *Samm50*-KI-ND, n = 13; WT-HFD, n = 15; *Samm50*-KI-HFD, n = 18; ∗*p* < 0.05; ∗∗*p* < 0.01; ∗∗∗*p* < 0.001; ∗∗∗∗*p* < 0.0001; n.s., not significant). **(E)** Representative images of H&E-stained liver sections (× 200 magnification). Scale bars: 10 μm. **(F)** Left: Representative Oil Red O-stained liver sections showing lipid accumulation (× 200 magnification). Scale bars: 100 μm. Right: Quantification of Oil Red O-positive areas in livers from HFD-fed WT and *Samm50*-KI mice (WT-HFD, n = 4; *Samm50*-KI-HFD, n = 4; ∗∗*p* < 0.01). **(G)** Hepatic TG levels of WT and *Samm50*-KI mice (WT-ND, n = 10; *Samm50*-KI-ND, n = 10; WT-HFD, n = 10; *Samm50*-KI-HFD, n = 12; ∗∗*p* < 0.01; ∗∗∗∗*p* < 0.0001; n.s., not significant). **(H)** Hepatic TC levels of WT and *Samm50*-KI mice (WT-ND, n = 10; *Samm50*-KI-ND, n = 10; WT-HFD, n = 10; *Samm50*-KI-HFD, n = 10; ∗*p* < 0.05; ∗∗∗*p* < 0.001; n.s., not significant). **(I)** Hepatic NEFA levels of WT and *Samm50*-KI mice (WT-ND, n = 10; *Samm50*-KI-ND, n = 10; WT-HFD, n = 10; *Samm50*-KI-HFD, n = 10; ∗∗∗*p* < 0.001; ∗∗∗∗*p* < 0.0001; n.s., not significant). **(J**, **K)** Serum TC (J) and ALT levels (K) (WT-ND, n ≥ 6; *Samm50*-KI-ND, n ≥ 7; WT-HFD, n ≥ 6; *Samm50*-KI-HFD, n ≥ 10; ∗*p* < 0.05; ∗∗∗*p* < 0.001; ∗∗∗∗*p* < 0.0001; n.s., not significant).

To investigate the contribution of SAMM50 D110G to MASLD development, we subjected *Samm50*-KI and WT mice to an HFD. *Samm50*-KI exhibited increased body weight during 16 weeks of HFD feeding, with significantly higher final body weight compared to WT (Figure 6A,B). Liver weight and liver-to-body weight ratios were also elevated in HFD-fed *Samm50*-KI mice (Figure 6C,D). Additionally, H&E and Oil Red O staining revealed pronounced hepatic lipid droplet accumulation in *Samm50*-KI following HFD (Figure 6E,F). Hepatic TG, TC, and NEFA levels were measured to quantitatively assess hepatic lipid accumulation. Under HFD conditions, *Samm50*-KI mice showed increased hepatic TG and TC levels compared with WT mice, while hepatic NEFA levels showed a slight increasing trend (Figure 6G–I). These findings support enhanced hepatic lipid accumulation in *Samm50*-KI mice. Serum TC and ALT levels were also increased in *Samm50*-KI mice (Figure 6J,K). Together, these results indicate that HFD promotes hepatic lipid accumulation and exacerbates liver injury in *Samm50*-KI mice, resulting in a more severe MASLD-like phenotype.

### 3.8 SAMM50 D110G promotes insulin resistance, glucose intolerance, and lipid accumulation following HFD

**Figure 7:**
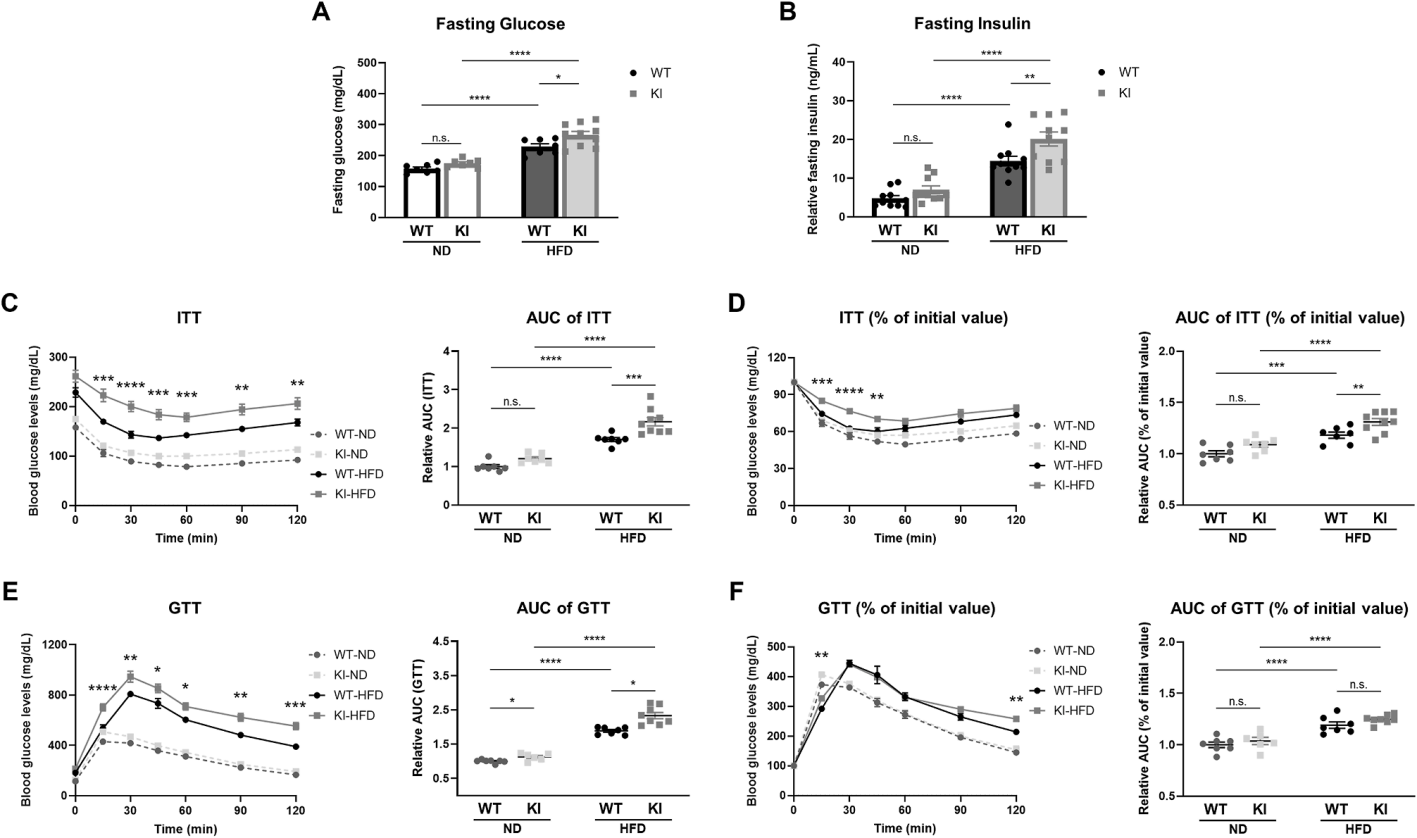
**Insulin resistance and glucose intolerance in HFD-fed *Samm50*-KI mice. (A)** Serum glucose levels (WT-ND, n = 7; *Samm50*-KI-ND, n = 7; WT-HFD, n = 7; *Samm50*-KI-HFD, n = 9; ∗*p* < 0.05; ∗∗∗∗*p* < 0.0001; n.s., not significant). **(B)** Serum insulin levels after 6 h of fasting (WT-ND, n = 10; *Samm50*-KI-ND, n = 10; WT-HFD, n = 10; *Samm50*-KI-HFD, n = 10; ∗∗*p* < 0.01; ∗∗∗∗*p* < 0.0001; n.s., not significant). **(C)** ITT and area under the curve (AUC) of ITT (WT-ND, n = 7; *Samm50*-KI-ND, n = 7; WT-HFD, n = 7; *Samm50*-KI-HFD, n = 9; ∗∗*p* < 0.01; ∗∗∗*p* < 0.001; ∗∗∗∗*p* < 0.0001; n.s., not significant). **(D)** ITT represented as % of initial glucose levels and corresponding AUC. **(E)** GTT and AUC of GTT (WT-ND, n = 7; *Samm50*-KI-ND, n = 6; WT-HFD, n = 7; *Samm50*-KI-HFD, n = 8; ∗*p* < 0.05; ∗∗*p* < 0.01; ∗∗∗*p* < 0.001; ∗∗∗∗*p* < 0.0001; n.s., not significant). **(F)** GTT represented as % of initial glucose levels and corresponding AUC.

To further assess systemic glucose homeostasis associated with MASLD progression, we performed GTT and ITT in both ND- and HFD-fed mice. HFD-fed WT and *Samm50*-KI mice exhibited higher serum glucose and insulin levels compared to ND-fed controls (Figure 7A,B). Notably, these levels were significantly elevated in *Samm50*-KI mice compared to WT mice. Following HFD feeding, both WT and *Samm50*-KI mice displayed insulin resistance and glucose intolerance compared to their ND-fed counterparts (Figure 7C–F). These metabolic abnormalities were more pronounced in HFD-fed *Samm50*-KI mice than in HFD-fed WT mice, indicating that the SAMM50 D110G mutation exacerbates insulin resistance and glucose intolerance under high-fat diet conditions.

We next investigated the molecular mechanisms underlying lipid accumulation, focusing on the involvement of mitochondria in cholesterol metabolism and lipogenesis [39,40]. Genes involved in mitochondrial structure and function, including *Samm50*, *Tom40*, *Opa1*, and *Pink1*, were downregulated in HFD-fed *Samm50*-KI compared with HFD-fed WT (Supplementary Fig. 10A). In association with the increase in hepatic and serum TC levels, we found an elevation in the expression of *Lss*, *Cyp51*, *Tm7sf2*, and *Dhcr24*, encoding proteins essential for cholesterol synthesis, in HFD-fed *Samm50*-KI (Supplementary Fig. 10B). Additionally, genes involved in de novo lipogenesis, including *Mlxipl*, *Srebf1*, *Acss2*, and *Fasn*, were upregulated in *Samm50*-KI (Supplementary Fig. 10C), indicating that mitochondrial dysfunction in *Samm50*-KI may promote hepatic lipogenesis. Taken together, these findings suggest that aberrant mitochondrial function resulting from the SAMM50 D110G mutation, together with HFD, promotes insulin resistance and lipogenesis, thereby driving MASLD progression.

## 4 Discussion

Several human genetic studies have demonstrated an association between *SAMM50* SNPs and MASLD [[4], [5], [6], [7], [8], [9], [10], [11]]. In line with previous studies, we demonstrated that *SAMM50* rs3761472 is significantly related to MASLD in clinical cohorts. Notably, rs3761472 is enriched in East Asian populations (Fig. 1E) and shows a significant association with MASLD in Chinese Han, Japanese, and Korean cohorts, indicating its potential relevance as a population-based biomarker [[5], [6], [7],^10^]. Also, studies utilizing *SAMM50* knockdown cells and *Samm50* knockout mice have shown that SAMM50 deficiency leads to increased lipid accumulation in the liver [8,41]. However, the molecular mechanisms underlying the association between the SNP rs3761472 in *SAMM50* and MASLD remain unclear.

To investigate these mechanisms in vivo, we established a murine model harboring the *SAMM50* rs3761472 variant using CRISPR/Cas9. Notably, a single amino acid substitution in SAMM50 resulted in profound effects in mice, impairing essential mitochondrial functions and lipid metabolism. Although missense mutations are not generally expected to alter transcript abundance [42,43], the SAMM50 D110G mutation resulted in reduced hepatic SAMM50 expression in *Samm50*-KI mice. Given the decreased expression of genes related to mitochondrial structure and function, D110G may alter retrograde signaling, a mitochondria-to-nucleus communication pathway through which mitochondrial dysfunction modulates nuclear gene transcription [44,45]. Supporting this notion, in vitro knockdown of SAMM50 decreased the mRNA expression of mitochondrial regulators such as MFN1, MFN2, and OPA1, further linking structural defects to transcriptional dysregulation [46]. ColabFold-based structural prediction comparing SAMM50 WT and D110G models suggested that the D110G substitution may induce a modest conformational change in a nearby loop region. To determine whether this change could affect SAMM50 interactions with MIC19 and MIC60 within the SAMM50–MIC19–MIC60 axis [13], we further performed AlphaFold-Multimer-based modeling of SAMM50 with MIC19 and MIC60, respectively. The predicted structures did not reveal substantial alterations in the overall complex formation, suggesting that the D110G variant is unlikely to markedly disrupt these interactions (Supplementary Fig. 11A–D). However, because the mitochondrial membrane localization of the D110G mutant protein was not directly examined in this study, potential effects of the variant on SAMM50 localization or membrane insertion cannot be excluded.

Interestingly, *SAMM50* knockdown enhances mitophagy in vitro [47], whereas *Samm50*-KI mice exhibited reduced mitophagy, suggesting distinct molecular consequences of the SAMM50 alteration. While *SAMM50* knockdown inhibits PINK1 processing and degradation, leading to its accumulation [47], the D110G mutation is likely to induce structural alterations in the SAM complex that impair OMM complex assembly or mitochondrial protein import, thereby preventing proper PINK1 stabilization on the OMM. Consistent with our observations, impaired mitophagy and decreased PINK1/Parkin expression have also been reported in the MASLD model [48].

The SAMM50 D110G mutation compromises ATP generation and promotes ROS production. Moreover, in HFD-fed *Samm50*-KI mice, hepatic steatosis, TG accumulation, and serum ALT levels were significantly increased, accompanied by insulin resistance and glucose intolerance. These in vivo observations align with our human genetic analyses, which demonstrated significant associations of rs3761472 with MASLD and related liver traits. Together, these integrated clinical and experimental findings highlight the critical role of mitochondrial integrity in MASLD development and offer valuable insights into its pathogenesis. Beyond MASLD, rs3761472 was also associated with increased risks of cirrhosis and hepatocellular carcinoma, suggesting broader implications in chronic liver disease. These findings support rs3761472 as a genetic biomarker for stratifying high-risk individuals and provide a rationale for future studies exploring therapeutic approaches aimed at improving mitochondrial function.

Although this study focused on the liver, previous studies have shown that mitochondrial gene deficiencies impair function in other tissues, particularly the brain and heart [35,49,50]. To explore potential extrahepatic effects of the SAMM50 D110G variant, we assessed *Samm50* expression in the brain, heart, kidney, and spleen. While expression was reduced in the brain and heart, no significant changes were detected in other tissues, and no downregulation of other mitochondrial genes was observed in any of the examined tissues (Supplementary Fig. 12A–D). These findings support the liver as the primary target organ, although potential effects in the brain and heart require further investigation.

In conclusion, our study establishes that a population-enriched missense variant in *SAMM50* exerts a direct functional impact on mitochondrial integrity and metabolic liver disease in vivo. By moving beyond statistical association to mechanistic validation, we demonstrate that the rs3761472 variant disrupts mitochondrial function and thereby contributes to MASLD susceptibility. These findings clarify the biological significance of a common *SAMM50* risk allele and provide a mechanistic basis for understanding how inherited variation in mitochondrial architecture influences metabolic liver disease, with potential implications for genetically informed risk assessment and targeted therapeutic strategies.

## CRediT authorship contribution statement

**Suyeon Kim:** Conceptualization, Data curation, Formal analysis, Investigation, Methodology, Validation, Visualization, Writing – original draft, Writing – review & editing. **Young Jin Kim:** Formal analysis, Investigation, Methodology. **Nahyun Kim:** Investigation, Validation. **Uijin Kim:** Investigation, Validation. **Yi Seul Park:** Formal analysis. **Jun Ho Yun:** Investigation. **Jiwon Heo:** Investigation. **Hyunwoo Lee:** Investigation. **Jiwon Choi:** Investigation. **Sinwoo Park:** Data curation, Investigation. **Inhae Jeong:** Investigation. **Bong-Jo Kim:** Conceptualization, Funding acquisition, Project administration, Resources, Supervision. **Ha Youn Shin:** Conceptualization, Funding acquisition, Project administration, Resources, Supervision, Writing – review & editing.

## DATA AVAILABILITY

The datasets generated and analyzed during the current study are available in the Korea National Institute of Health PheWEB site (https://coda.nih.go.kr/usab/pheweb/intro.do), the BioBank Japan PheWeb site (https://pheweb.jp), and the UK Biobank PheWeb site (https://pheweb.org/UKB-TOPMed/). The datasets used and analyzed during the current study are available from the corresponding author on reasonable request.

## Funding

This work was supported by the National Research Foundation of Korea (NRF) grant funded by the Korean Government (MSIT) (2022R1F1A10733251220682073250102), National Institute of Health, Republic of Korea (2016-NI73001-02, 2022-NI-067-02), and Basic Science Research Program through the National Research Foundation of Korea (NRF) funded by the Ministry of Education (RS-2024-00460628). This paper was also written as part of Konkuk University's research support program for faculty on sabbatical leave in 2024.

## Declaration of competing interest

The authors declare that they have no known competing financial interests or personal relationships that could have appeared to influence the work reported in this paper.

## Data Availability

Data will be made available on request.
