# Supplementary material for: SAMM50 rs3761472 causes mitochondrial dysfunction and progression of metabolic dysfunction-associated steatotic liver disease
Source: Mol Metab. 2026 Jul 17;111:102423. doi: 10.1016/j.molmet.2026.102423 (PMC13446200; doi:10.1016/j.molmet.2026.102423)
Supplement: Supplementary file 1 [file mmc1.pdf]

Supplementary data

SAMM50 rs3761472 causes mitochondrial dysfunction and progression of metabolic dysfunction-associated steatotic liver disease

Suyeon Kim *et al.*

MATERIALS AND METHODS

Transmission electron microscopy (TEM)

Mouse liver tissues were harvested and cut into approximately 1 mm^3 pieces. Subsequent sample processing and transmission electron microscopy were performed by TEMES Co., Ltd. (Seongnam, Gyeonggi-do, South Korea). Specimens were fixed in 2% glutaraldehyde and 2% paraformaldehyde prepared in 0.1 M phosphate buffer (pH 7.4) for 24 h at 4°C. After washing with 0.1 M phosphate buffer, the specimens were post-fixed in 1% OsO_4 prepared in 0.1 M phosphate buffer for 1.5 h. The specimens were then dehydrated through a graded ethanol series (50–100%), infiltrated with propylene oxide for 10 min, and embedded in Poly/Bed 812 resin (Polysciences, USA) for 24 h. Ultrathin sections were cut at 70 nm using an Ultracut UCT ultramicrotome (Leica, Germany) with a diamond knife and stained with 5% uranyl acetate and 1% lead citrate. The sections were examined using a transmission electron microscope (JEM-1011, JEOL, Japan) at an accelerating voltage of 80 kV and were photographed with a digital CCD camera (EMSIS GmbH, Muenster, Germany).

A

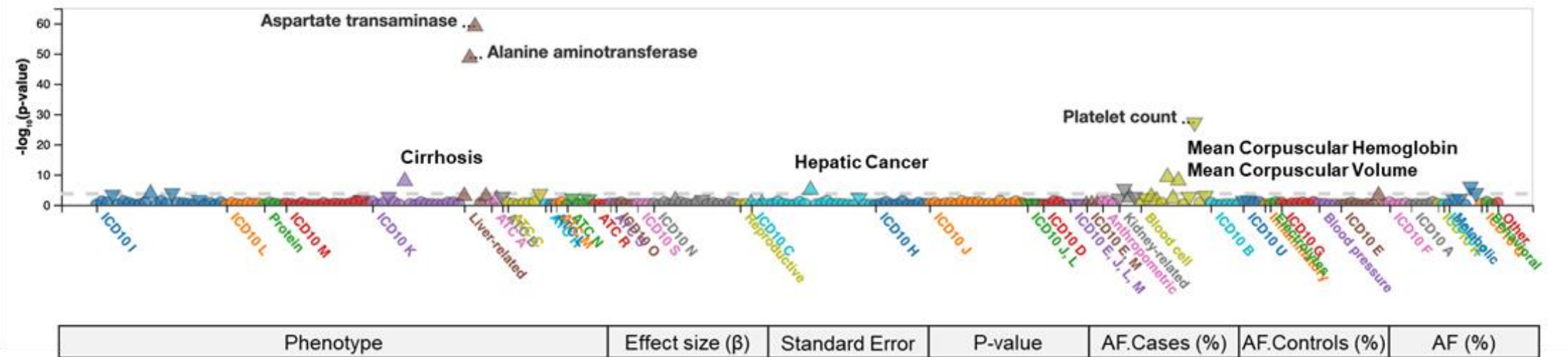

Phenotype	Effect size (β)	Standard Error	P-value	AF.Cases (%)	AF.Controls (%)	AF (%)
Cirrhosis	0.1635	0.0287	1.19E-08	46.0	42.1	42.2
Hepatic Cancer	0.1395	0.0316	1.00E-05	45.4	42.1	42.2
ALT	0.0506	0.0034	2.60E-49	-	-	42.0
AST	0.0572	0.0035	1.10E-59	-	-	42.0

AF.Cases: Allele Frequency in Cases
AF.Controls: Allele Frequency in Controls
AF: Overall Allele Frequency

B

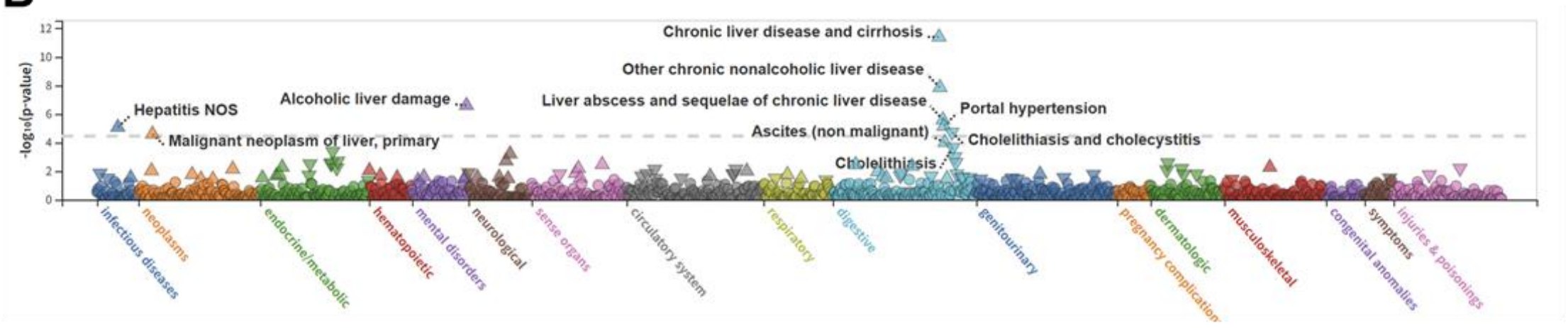

Phenotype	Effect size (β)	Standard Error	P-value	AF.Cases (%)	AF.Controls (%)	AF (%)
Chronic liver disease and cirrhosis	0.26	0.037	4.10E-12	19	15	16
Other chronic nonalcoholic liver disease	0.28	0.049	1.40E-08	19	15	16
Alcoholic liver damage	0.36	0.069	2.50E-07	20	16	16
Liver abscess and sequelae of chronic liver disease	0.3	0.064	2.60E-06	19	15	16
Portal hypertension	0.39	0.087	7.20E-06	20	15	16
Hepatitis NOS	0.43	0.097	8.40E-06	21	16	16
Malignant neoplasm of liver, primary	0.68	0.16	2.50E-05	24	16	16

AF.Cases: Allele Frequency in Cases
AF.Controls: Allele Frequency in Controls
AF: Overall Allele Frequency

Supplementary Figure 1. PheWAS plots of *SAMM50* rs3761472 associations in the Biobank Japan and UK Biobank cohorts.

(A) PheWAS plot of *SAMM50* rs3761472 in the Biobank Japan cohort, highlighting representative top associations (upper panel). Summary table of the significant phenotypes in Biobank Japan, including effect size (β), standard error (SE), *P*-value, and allele frequencies in cases, controls, and the overall population (lower panel). **(B)** PheWAS plot of *SAMM50* rs3761472 in the UK Biobank cohort, showing representative associations (upper panel). Corresponding summary table of significant traits in the UK Biobank cohort (lower panel).

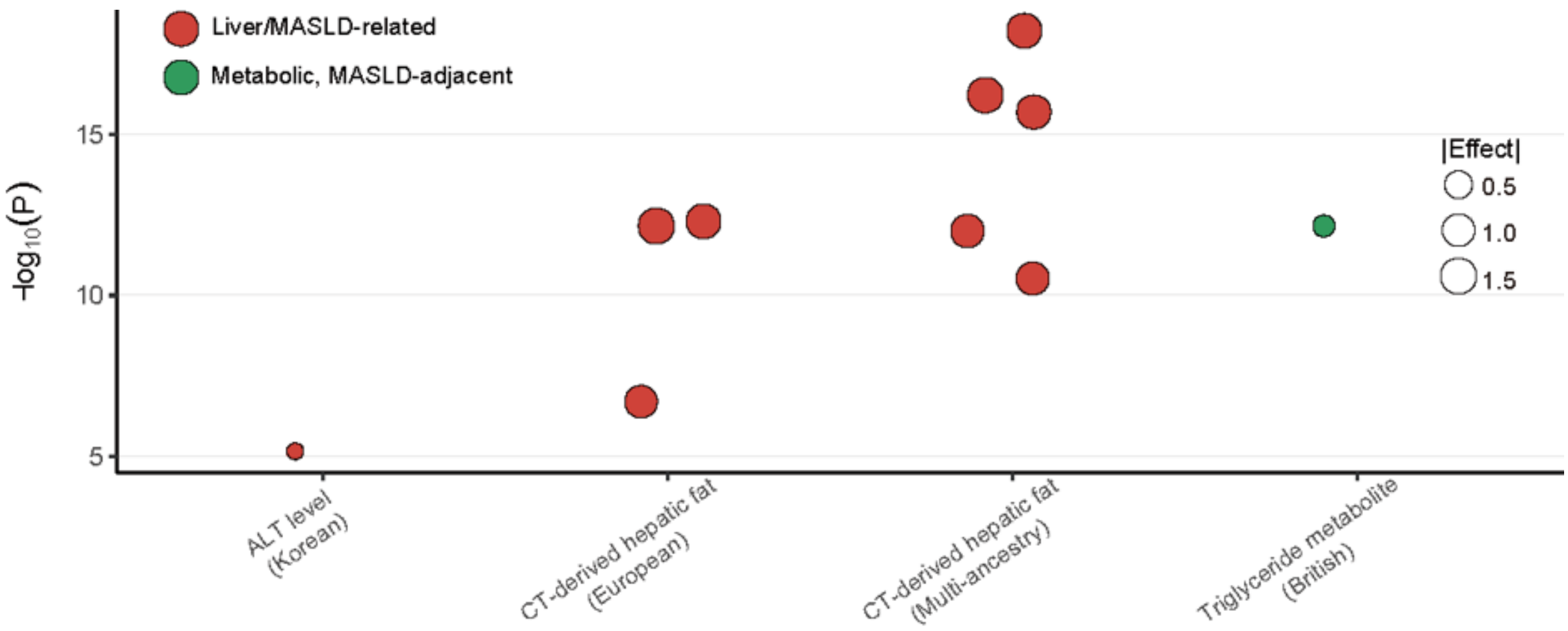

Supplementary Figure 2. GWAS Catalog annotations of rs3761472-associated traits. Dot plot showing previously reported GWAS associations of rs3761472 with liver/MASLD-related and metabolic MASLD-adjacent traits. The y-axis represents $-\log_{10}(P)$, and dot size indicates the absolute effect size. Red dots indicate liver/MASLD-related traits, including alanine aminotransferase (ALT) levels and computed tomography (CT)-derived hepatic fat, while the green dot indicates a triglyceride-related metabolic trait.

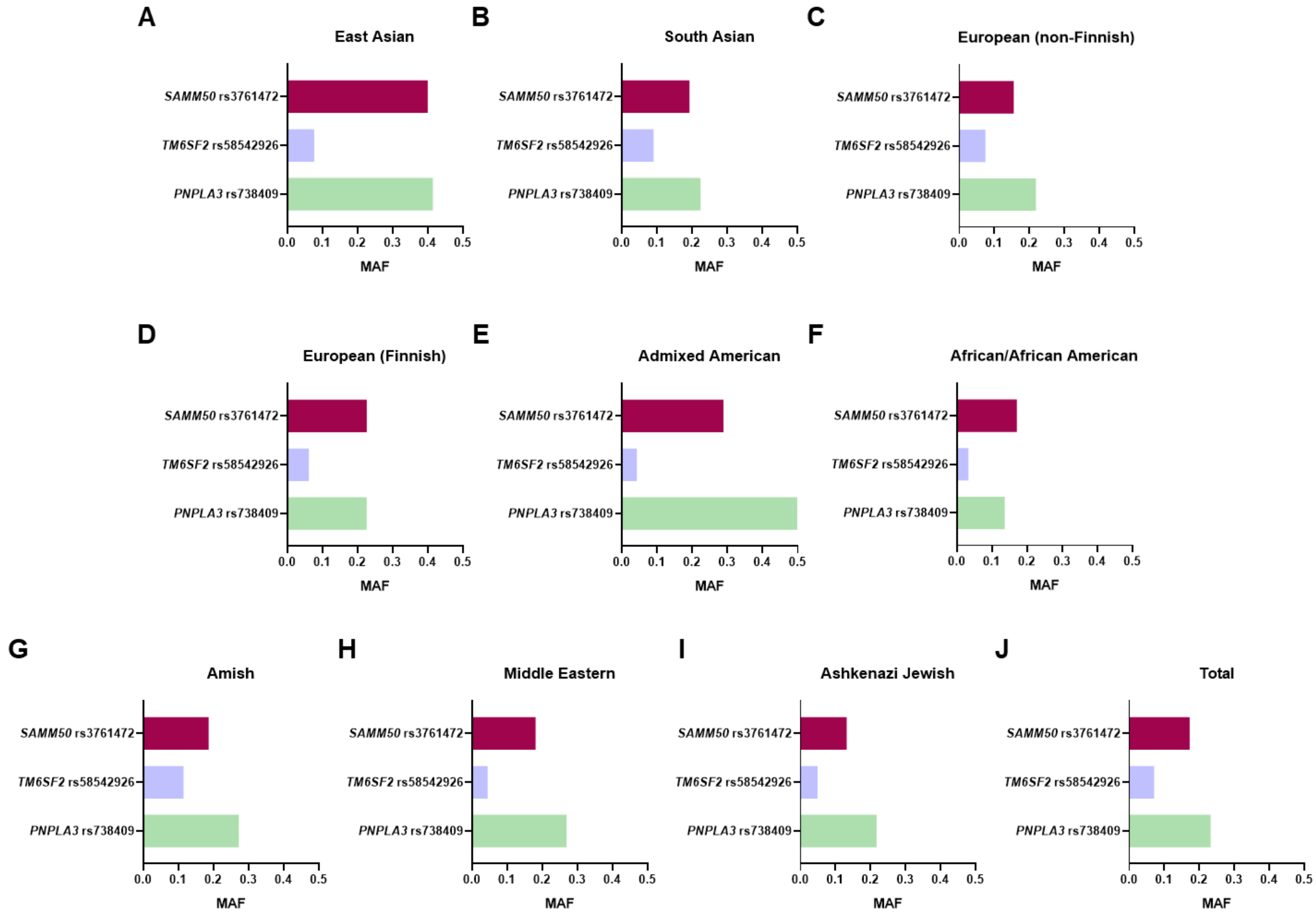

Supplementary Figure 3. Minor allele frequencies (MAFs) of MASLD-associated SNPs across populations.

(A–J) MAFs of *SAMM50* rs3761472, *TM6SF2* rs58542926, and *PNPLA3* rs738409 in East Asian (A), South Asian (B), European (non-Finnish) (C), European (Finnish) (D), Admixed American (E), African/African American (F), Amish (G), Middle Eastern (H), Ashkenazi Jewish (I), and overall (J) populations.

A

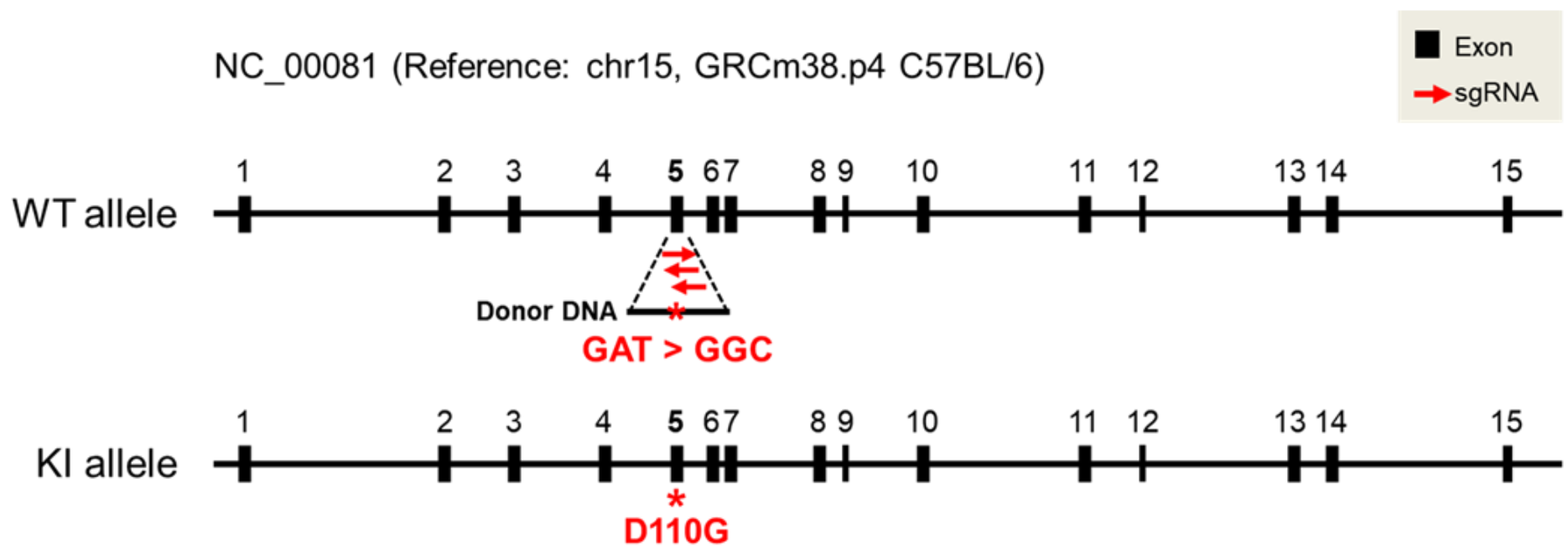

B

Target locus	Target sequence for sgRNA	Donor sequence
Exon 5	GTGAAGATGCCCTGCCCAATGGG	CGGAGGACAGTTTCCAGGAGTCAGTGGGTCCTGGGA ACGTACCCAGCCTGGCAGGCATGGTGGCAGTGCCTT TACCCCCGAGCGGTGTTGACCCTTTTGATACTTACTTC TTTAGGTGAAGGCGCCTTACCTAATGGGTTAGATGTCA CCTTTGAAGTGACAGAGCTGAGGAGACTGACGGGC
	GGGACGGGTTACCCAATCTACAG	
	GGTTACCCAATCTACAGTGGAAA	
Green, PAM; Red, mutant nucleotide (GAT > GGC); Blue, Silent mutation (CTG > TTA, CCC > CCT)		

Supplementary Figure 4. Experimental design for generating SAMM50 D110G mutant mice using the CRISPR/Cas9 system.

(A) Genomic locus of the mouse *Samm50* gene showing the sgRNA target sites in the target region. In the knock-in mice, the aspartic acid residue at position 110 (D110), encoded by exon 5 of *Samm50*, was replaced with glycine (G110). **(B)** sgRNA target sequences and the donor sequence used to introduce the *Samm50* D110G mutation. The protospacer-adjacent motif (PAM) sequences are highlighted in green, target nucleotide substitutions are shown in red, and silent mutations are marked in blue. To introduce the D110G mutation, the original GAT codon in exon 5 of *Samm50* was replaced with GGC. Two additional silent mutations, CTG to TTA and CCC to CCT, were introduced into the adjacent target sequence.

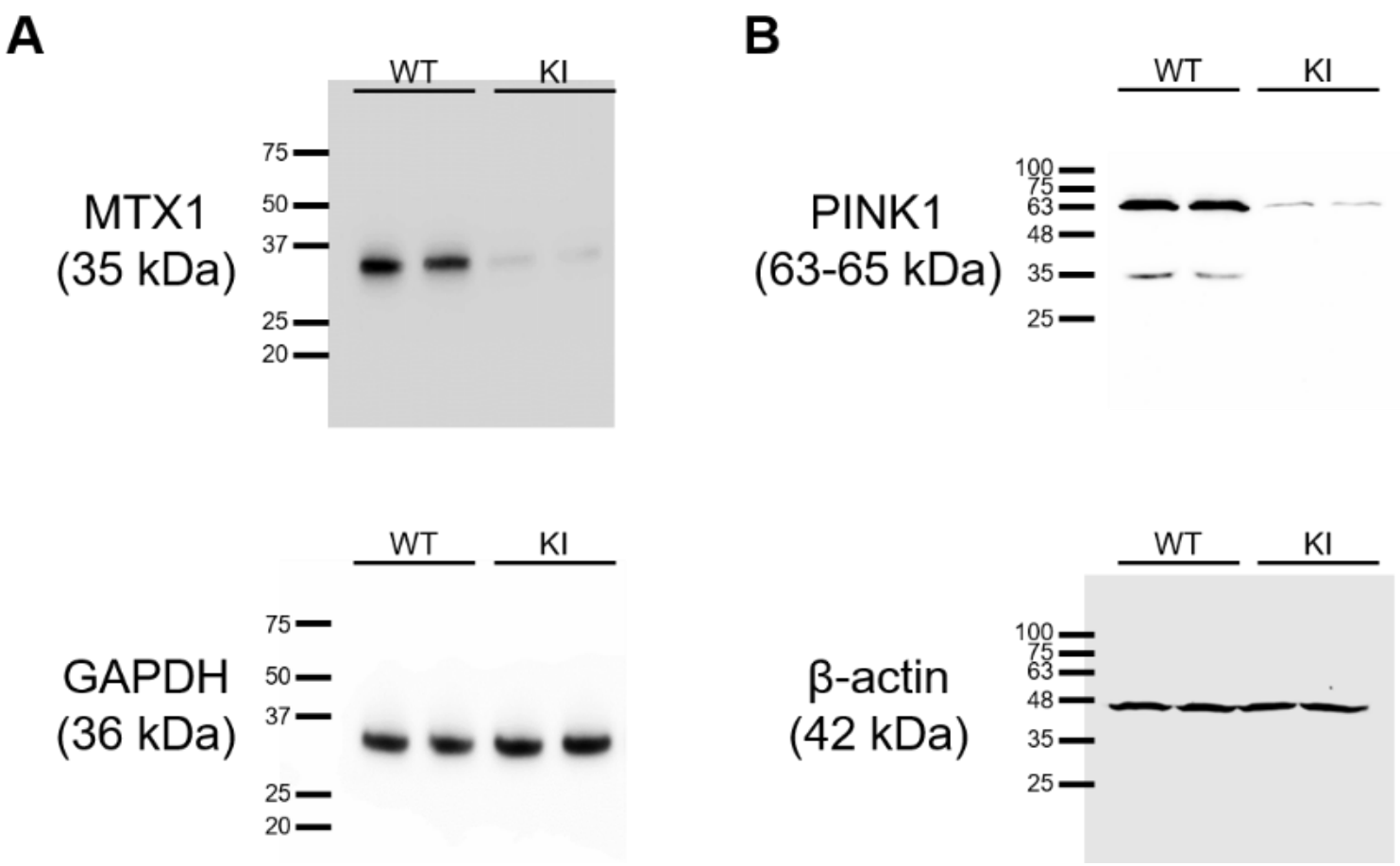

Supplementary Figure 5. Unedited western blot images of MTX1 and PINK1.
(A) MTX1 and **(B)** PINK1.

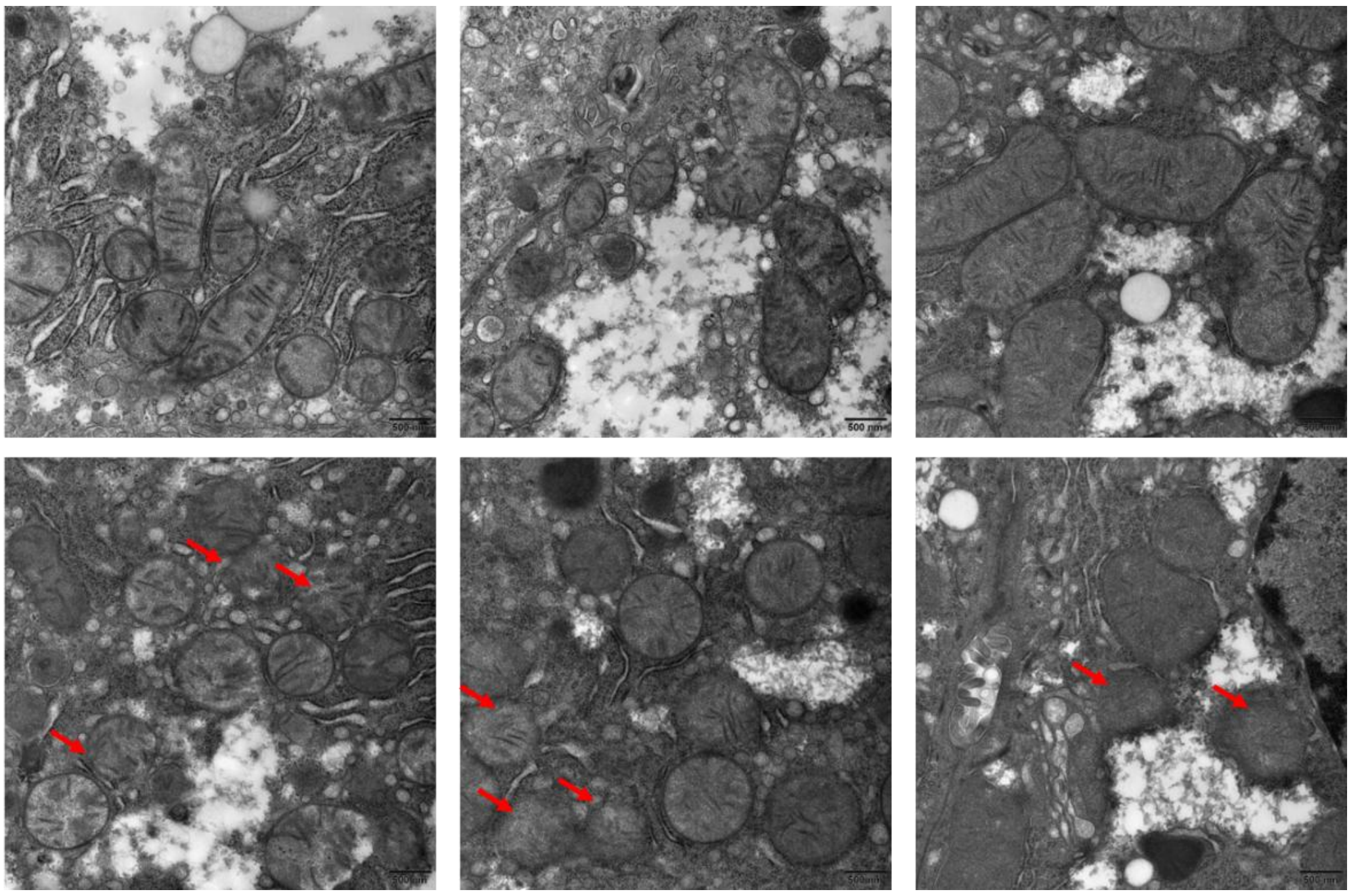

Supplementary Figure 6. Representative transmission electron microscopy (TEM) images of liver tissues from WT and *Samm50*-KI mice.

TEM analysis revealed mitochondrial ultrastructural abnormalities in the livers of *Samm50*-KI mice compared with WT controls, including disrupted mitochondrial morphology (×30,000 magnification). Scale bars: 500 nm. Red arrows indicate disrupted mitochondrial membranes.

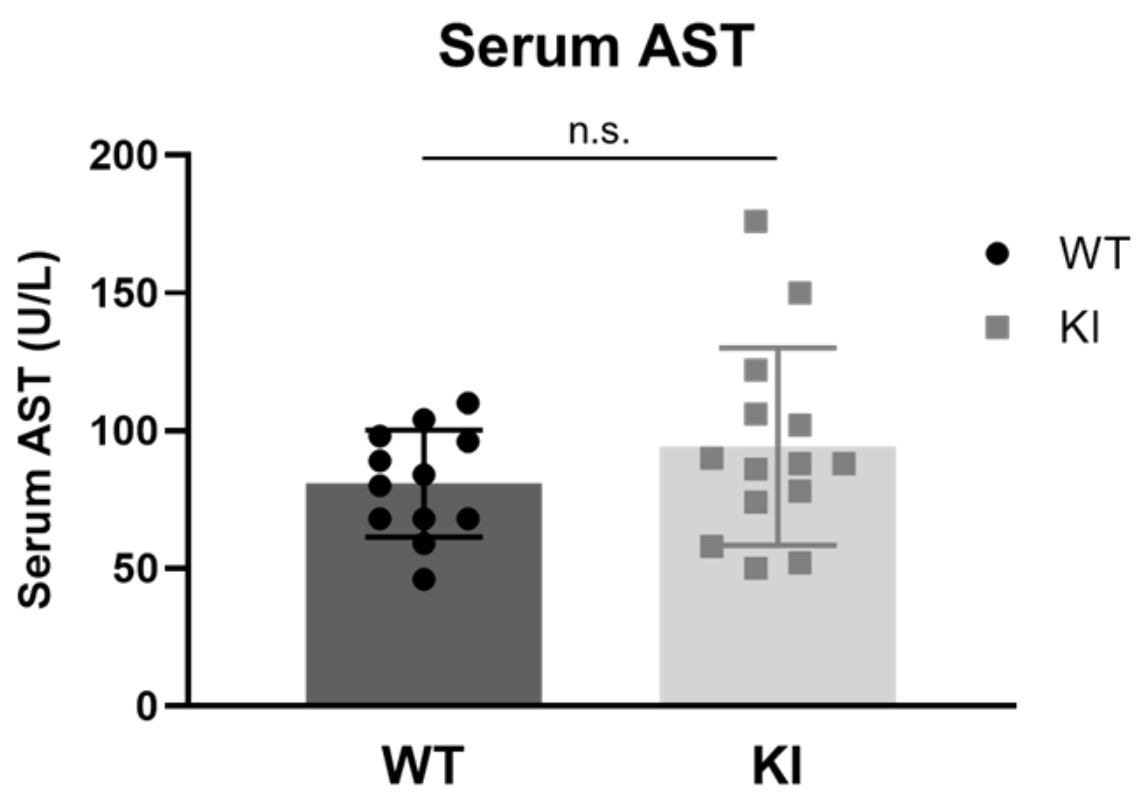

Supplementary Figure 7. Serum aspartate aminotransferase (AST) levels in WT and *Samm50*-KI mice.

Serum AST levels were measured in WT and *Samm50*-KI mice. Data are presented as means ± SEM (WT, n = 12; *Samm50*-KI, n = 14; n.s., not significant).

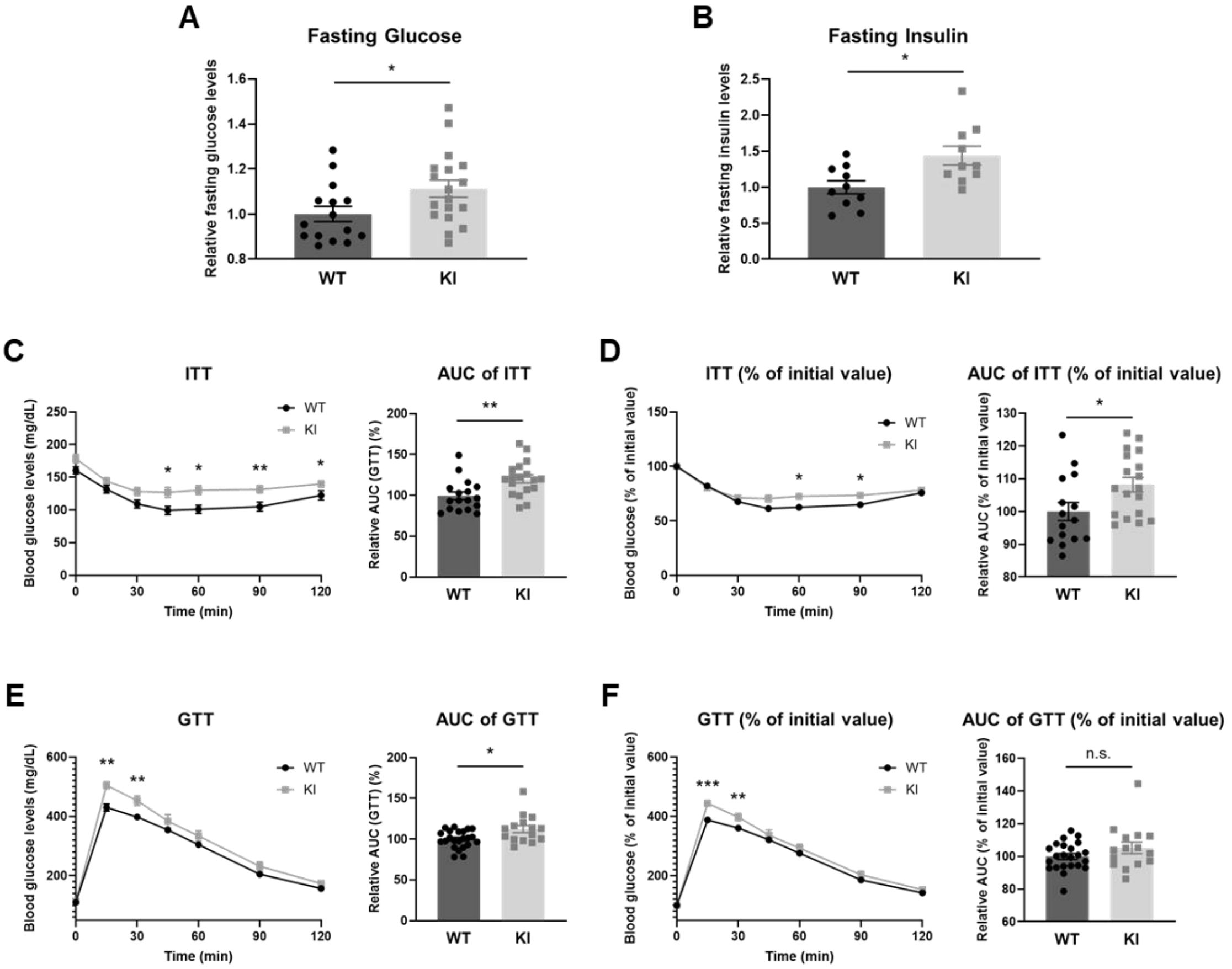

Supplementary Figure 8. SAMM50 D110G leads to insulin resistance and glucose intolerance in mice.

(A) Serum glucose levels (WT, n = 15; *Samm50*-KI, n = 18; $*p < 0.05$). **(B)** Serum insulin levels after 6 hours of fasting in WT and *Samm50*-KI mice (WT, n = 10; *Samm50*-KI, n = 10; $*p < 0.05$). **(C)** ITT and area under the curve (AUC) of ITT (WT, n = 15; *Samm50*-KI, n = 18; $*p < 0.05$; $**p < 0.01$). **(D)** ITT represented as % of initial glucose levels and the corresponding AUC (WT, n = 15; *Samm50*-KI, n = 18; $*p < 0.05$). **(E)** GTT and AUC of GTT (WT, n = 23; *Samm50*-KI, n = 15; $*p < 0.05$; $**p < 0.01$). **(F)** GTT represented as % of initial glucose levels and the corresponding AUC (WT, n = 23; *Samm50*-KI, n = 15; $**p < 0.01$; $***p < 0.001$; n.s., not significant). Data are presented as means ± SEM.

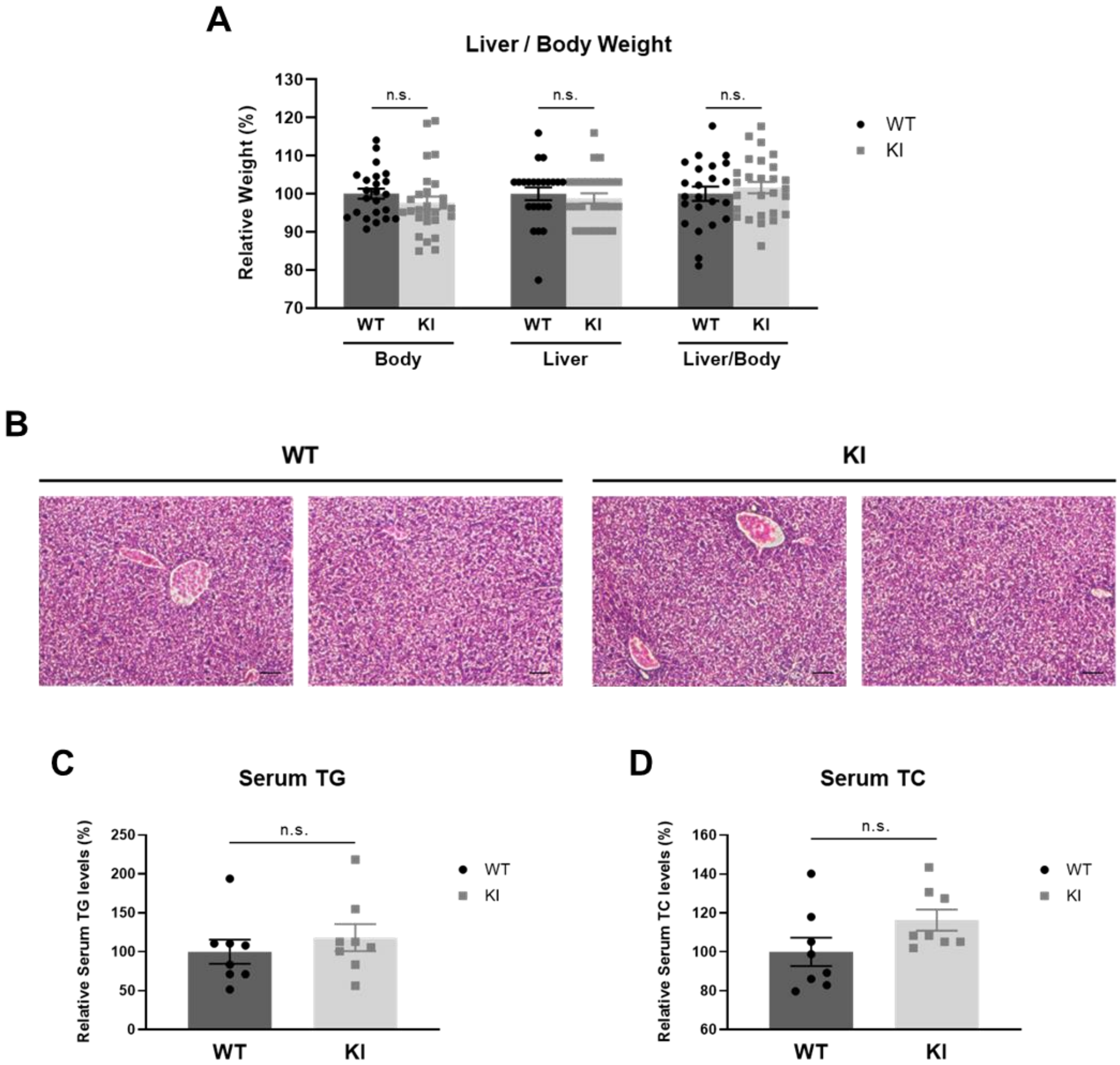

Supplementary Figure 9. Examination of MASLD features in *Samm50*-KI mice.

(A) Comparison of body weights, liver weights, and liver-to-body weight ratios between WT and *Samm50*-KI mice (WT, n = 23; *Samm50*-KI, n = 27; n.s., not significant). **(B)** Representative images of H&E-stained liver sections from WT and *Samm50*-KI mice (×100 magnification). Scale bars: 10 μm. **(C, D)** Relative levels of serum triglycerides (TG) (C) and total cholesterol (TC) (D) in WT and *Samm50*-KI mice (WT, n = 8; *Samm50*-KI, n = 8; n.s., not significant). Data are presented as means ± SEM.

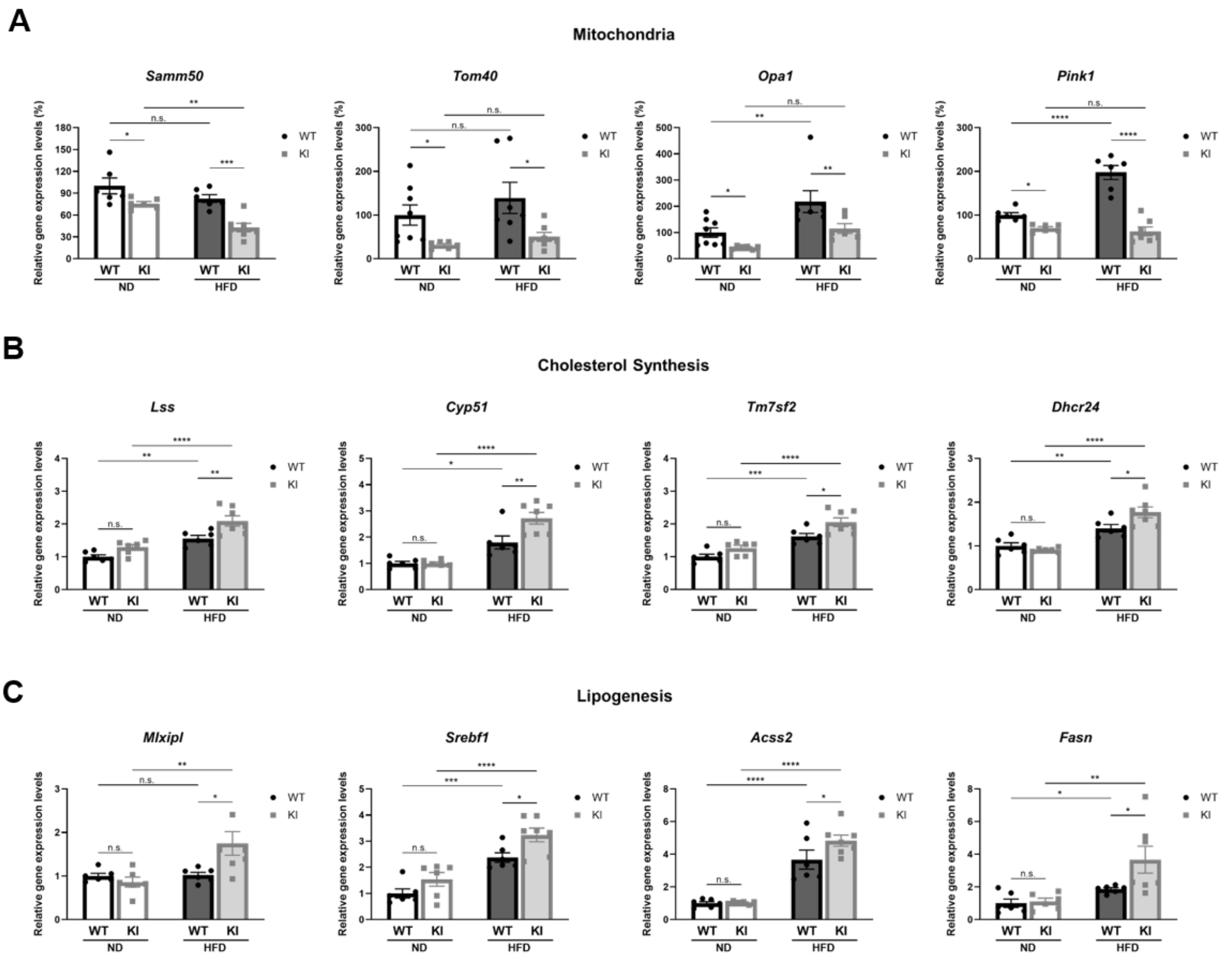

Supplementary Figure 10. Downregulation of mitochondria-associated genes promotes cholesterol synthesis and liver lipogenesis in HFD-fed *Samm50*-KI mice.

(A) Relative expression levels of the mitochondrial structure- and function-related genes, *Samm50*, *Tom40*, *Opa1*, and *Pink1*. **(B)** Relative expression levels of the cholesterol synthesis pathway-related genes, *Lss*, *Cyp51*, *Tm7sf2*, and *Dhcr24*. **(C)** Relative expression levels of the de novo lipogenesis-related genes, *Mlxipl*, *Srebf1*, *Acss2*, and *Fasn*. The mRNA level of each gene was normalized to that of *Gapdh* and expressed relative to the corresponding level in ND-fed WT mice (WT-ND, $n \geq 6$; *Samm50*-KI-ND, $n \geq 6$; WT-HFD, $n \geq 6$; *Samm50*-KI-HFD, $n \geq 6$). Results are presented as means ± SEM (*$p < 0.05$; **$p < 0.01$; ***$p < 0.001$; ****$p < 0.0001$; n.s., not significant).

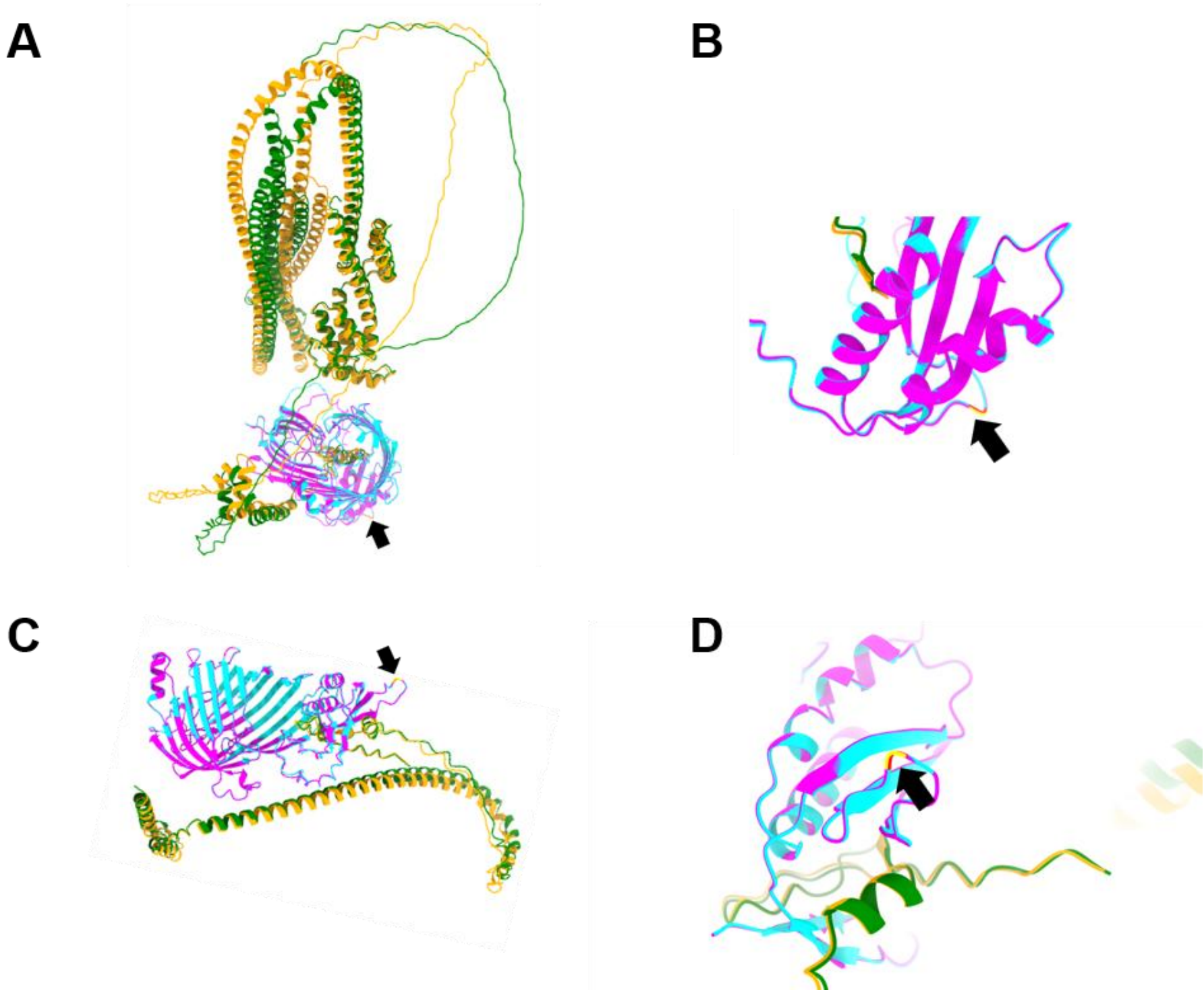

Supplementary Figure 11. Structural comparison of WT and D110G SAMM50–MIC60 and SAMM50–MIC19 complexes predicted by AlphaFold-Multimer.

(A) Global structural overlay of the human WT and D110G SAMM50–MIC60 complexes. WT SAMM50 and MIC60 are shown in cyan and green, respectively, whereas D110G SAMM50 and MIC60 are shown in magenta and orange, respectively. Residue D110 in the WT model and G110 in the mutant model are highlighted in red and yellow, respectively, and indicated by a black arrow. **(B)** Local structural view of the region surrounding residue 110 in the WT and D110G SAMM50–MIC60 models. **(C)** Global structural overlay of the human WT and D110G SAMM50–MIC19 complexes. WT SAMM50 and MIC19 are shown in cyan and green, respectively, whereas D110G SAMM50 and MIC19 are shown in magenta and orange, respectively. Residue D110 in the WT model and G110 in the mutant model are highlighted in red and yellow, respectively, and indicated by a black arrow. **(D)** Local structural view of the region surrounding residue 110 in the WT and D110G SAMM50–MIC19 models.

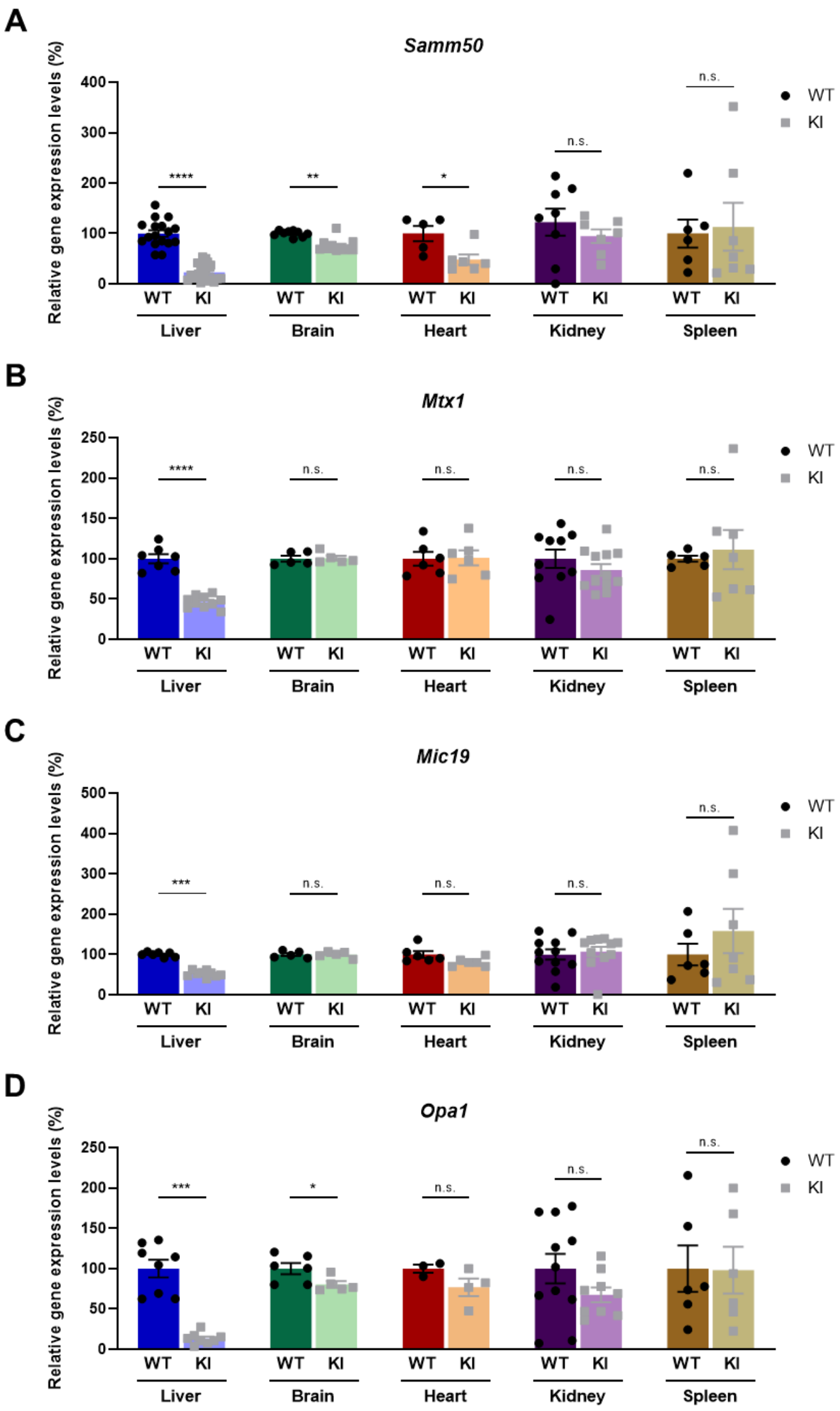

Supplementary Figure 12. Comparative expression of mitochondria-associated genes across multiple tissues in WT and *Samm50*-KI mice.

(A–D) Relative mRNA expression levels of mitochondria-related genes: *Samm50* (A), *Mtx1* (B), *Mic19* (C), and *Opa1* (D) in the liver, brain, heart, kidney, and spleen of WT and *Samm50*-KI mice. Gene expression was normalized to *Gapdh* and presented relative to WT levels (WT, n ≥ 5; *Samm50*-KI, n ≥ 5). Data are shown as means ± SEM (*$p < 0.05$; **$p < 0.01$; ***$p < 0.001$; ****$p < 0.0001$; n.s., not significant).

Supplementary Table 1. In silico predictions of the functional impact of *SAMM50* rs3761472

Algorithm	Predicted effect	Homepage
FATHMM	Tolerated	http://fathmm.biocompute.org.uk/
MutationAssessor	Low	http://mutationassessor.org/r3/
SIFT	**Damaging**	https://sift.bii.a-star.edu.sg/
M-CAP	Benign	http://bejerano.stanford.edu/mcap/
MutationTester	**Disease-causing**	http://mutationtaster.org/
PolyPhen-2	**Damaging**	http://genetics.bwh.harvard.edu/pph2/
PROVEAN	Neutral	http://provean.jcvi.org/index.php
CADD	**18.1**	https://cadd.gs.washington.edu/

Supplementary Table 2. Primer sequences used for PCR-based genotyping of the *Samm50* gene

Target gene		Primer sequences
Samm50	Forward	5'-AGGACAGAGAGCCTAGTCGG-3'
	Reverse	5'-TCAGGCTGACCAGTCCTAGG-3'

Supplementary Table 3. Gene-specific primer sequences for RT-qPCR analysis

Target gene		Primer sequences
Gapdh	Forward	5'-TGTGTCCGTCGTGGATCTGA-3'
	Reverse	5'-TTGCTGTTGAAGTCGCAGGAG-3'
Samm50	Forward	5'-AAACCTCATTGAGGTAATGC-3'
	Reverse	5'-CAGCTCTGTCACTTCAAAGG-3'
Dnajc11	Forward	5'-GAACGAACCCCAAGGGAACC-3'
	Reverse	5'-TTCTGGGTGGAGAGACTTCC-3'
Mic19	Forward	5'-CAGAGACGCCTTAAGCAAGC-3'
	Reverse	5'-AGCTGCTCTTTGTAGAATGC-3'
Mic60	Forward	5'-AGTTCTGGGTTGACTGCTGG-3'
	Reverse	5'-TTAGTGGACCAGACTGAACC-3'
Mtx1	Forward	5'-CCTTCAGGAACTCTGCCTGC-3’
	Reverse	5'-GCAGTAGTTTCTCCTCCAGC-3’
Mtx2	Forward	5'-GACTGCAGAGCTGTATCTCC-3’
	Reverse	5'-GGTCTACATCTTCTAAGACC-3’
Tom40	Forward	5'-AAGTGCAAGGAGCTGTTTCC-3’
	Reverse	5'-CCACTATTGTCCATGTCACC-3’
Tom70	Forward	5'-GAGTGTTTAGAAGACGTCAC-3’
	Reverse	5'-TCAGAGGTTCACGGTTCTTG-3’
Opa1	Forward	5'-CTGCAGAAGGACGACAAAGG-3’
	Reverse	5'-CCTGAGCAATCATTTCCAGC-3’
Mfn1	Forward	5'-CCTGTCCAGGTGCATAATGG-3’
	Reverse	5'-ACCAGATCATCCTCGGATGC-3’
Mfn2	Forward	5'-GGTTTATAAGAACGAGCTGC-3’
	Reverse	5'-AATTCAGGTCATAGCTGAGG-3’
Dnm1l	Forward	5'-CTGAAGGCATTACAAGGAGC-3'
	Reverse	5'-CGAGCAAATTCATACACTGC-3'
Pink1	Forward	5'-ATGTGGAATATCTCGGCAGG-3'
	Reverse	5'-CGGATGATGTTAGGGTGTGG-3'
Prkn	Forward	5'-CCAGAGGAAAGTCACCTGCGAA-3'
	Reverse	5'-GTTCGAGCAGTGAGTCGCAATC-3'
Atp5a1	Forward	5'-TGGTGAAGAGACTGACGGATGC-3'
	Reverse	5'-TCAAAGCGTGCTTGCCGTTGTC-3'

Atp5b	Forward	5'-CTCTGACTGGTTTGACCGTTGC-3'
	Reverse	5'-TGGTAGCCTACAGCAGAAGGGA-3'
Atp5c1	Forward	5'-CCGAGTGTATGGGACAGGTTCT-3'
	Reverse	5'-GGAGTGAATAGCACCACAAAGCC-3'
Atp5d	Forward	5'-ACTGGAGCCTTTGGCATCTTGG-3'
	Reverse	5'-AGTCGGCATTCACAGTGACGGA-3'
Atp5g2	Forward	5'-TGCAGTGGAGTTGAAGCGACCA-3'
	Reverse	5'-GCTGGTTTGGAAACTGCGGCTA-3'
Sirt3	Forward	5'-CCTGCAAGGTTCCTACTCCA-3'
	Reverse	5'-AGCCTTTCCACACCATGAAC-3'
Sod2	Forward	5'-CCAGACCTGCCTTACGACTA-3'
	Reverse	5'-TGAAGAGCGACCTGAGTTGT-3'
Prdx1	Forward	5'-TGCCAAGTGATTGGCGCTTCTG-3’
	Reverse	5'-AGCAATGGTGCGCTTGGGATCT-3’
Prdx6	Forward	5'-ATCCGCTTCCACGATTTCCTGG-3'
	Reverse	5'-TTCCTCTTGGCAAACTCTGGCG-3'
Gpx1	Forward	5'-CGCTCTTTACCTTCCTGCGGAA-3'
	Reverse	5'-AGTTCCAGGCAATGTCGTTGCG-3'
Ucp2	Forward	5'-TAAAGGTCCGCTTCCAGGCTCA-3'
	Reverse	5'-ACGGGCAACATTGGGAGAAGTC-3'
Nfkb2	Forward	5'-CTGGTGGACACATACAGGAAGAC-3'
	Reverse	5'-ATAGGCACTGTCTTCTTTCACCTC-3'
Relb	Forward	5'-CTTTGCCTATGATCCTTCTGC-3'
	Reverse	5'-GAGTCCAGTGATAGGGGCTCT-3'
Il6	Forward	5'-AGTTGCCTTCTTGGGACTGA-3'
	Reverse	5'-TCCACGATTTCCCAGAGAAC-3'
Tnfa	Forward	5'-AGCCCCCAGTGTGTATCCTT-3'
	Reverse	5'-ACAGTCCAGGTCACTGTCCC-3'
Il1b	Forward	5'-CTTCAGGCAGGCAGTATCACTC-3'
	Reverse	5'-TTGTTGTTCATCTCGGAGCC-3'
Ifna	Forward	5'-GGACTTTGGATTCCCGCAGGAGAAG-3'
	Reverse	5'-GCTGCATCAGACAGCCTTGCAGGTC-3'
Ifnb	Forward	5'-AACCTCACCTACAGGGCGGACTTCA-3'
	Reverse	5'-TCCCACGTCAATCTTTCCTCTTGCTTT-3'
Ifng	Forward	5'-TCAAGTGGCATAGATGTGGAAGAA-3'
	Reverse	5'-TGGCTCTGCAGGATTTTCATG-3'

Nos2	Forward	5'-CAGAGGACCCAGAGACAAGC-3'
	Reverse	5'-TGCTGAAACATTTCCTGTGC-3'
Cox2	Forward	5'-CAGACAACATAAACTGCGCCTT-3'
	Reverse	5'-GATACACCTCTCCACCAATGACC-3'
Bax	Forward	5'-AGCAAACTGGTGCTCAAGGC-3'
	Reverse	5'-CCACAAAGATGGTCACTGTC-3'
Bcl2	Forward	5'-GTGGTGGAGGAACTCTTCA-3'
	Reverse	5'-GTTCCACAAAGGCATCCCA-3'
Lss	Forward	5'-GCACACCACAGACCTGAGTTTC-3'
	Reverse	5'-CAGTGTGCTGAAGGAGAAACCAC-3'
Cyp51	Forward	5'-ATCCAGAAGCGCAGGCTGTCAA-3'
	Reverse	5'-CAGTCCGATGAGCATCCCTGAT-3'
Tm7sf2	Forward	5'-GGTCAATGGCTTCCAGTTGCTG-3'
	Reverse	5'-CTCCAAAGACCAGCATGAAGCC-3'
Dhcr24	Forward	5'-CATCTTCCGCTACCTCTTCG-3'
	Reverse	5'-CTCTGCTTCATCTCCCTTGG-3'
Mlxipl	Forward	5'-CGACACTCACCCACCTCTTC-3'
	Reverse	5'-TTGTTCAGCCGGATCTTGTC-3'
Srebf1	Forward	5'-CGACTACATCCGCTTCTTGCAG-3'
	Reverse	5'-CCTCCATAGACACATCTGTGCC-3'
Acss2	Forward	5'-ACTTGGCGACAAAGTTGCTTTT-3'
	Reverse	5'-TCTGAACACCCTGTTTACGGAG-3'
Fasn	Forward	5'-TGGGTTCTAGCCAGCAGAGT-3'
	Reverse	5'-ACCACCAGAGACCGTTATGC-3'

Supplementary Table 4. Primary antibodies used for western blotting

Antibody	Host	Manufacturer	Catalog number
Anti-GAPDH	Mouse	Santa Cruz	sc-47724
Anti-β-actin	Rabbit	Abcam	ab8227
Anti-SAMM50	Rabbit	Abcam	ab133709
Anti-MTX1	Rabbit	Abcam	ab233205
Anti-PINK1	Rabbit	Abcam	ab23707
Anti-p62	Rabbit	Cell signaling Technology	5114
Anti-LC3B	Rabbit	Cell signaling Technology	2775
Anti-ATG5	Rabbit	Cell signaling Technology	12994
